# A nuclear DNA barcode for eastern North American oaks and application to a study of hybridization in an Arboretum setting

**DOI:** 10.1002/ece3.4122

**Published:** 2018-05-08

**Authors:** Elisabeth Fitzek, Adline Delcamp, Erwan Guichoux, Marlene Hahn, Matthew Lobdell, Andrew L. Hipp

**Affiliations:** ^1^ Herbarium The Morton Arboretum Lisle Illinois; ^2^ Site de Pierroton, Platforme Genome Transcriptome INRA CESTAS France; ^3^ UMR1202 Biodiversité Gènes and Communautés University of Bordeaux CESTAS France; ^4^ Department of Botany The Field Museum Chicago Illinois; ^5^ Present address: Department of Biological Sciences Northern Illinois University DeKalb Illinois

**Keywords:** barcoding, hybridization, North American white oaks, RAD‐seq, SNP barcode

## Abstract

DNA barcoding has proved difficult in a number of woody plant genera, including the ecologically important oak genus *Quercus*. In this study, we utilized restrictionsite‐associated DNA sequencing (RAD‐seq) to develop an economical single nucleotide polymorphism (SNP) DNA barcoding system that suffices to distinguish eight common, sympatric eastern North American white oak species. Two de novo clustering pipelines, PyRAD and *Stacks*, were used in combination with postclustering bioinformatic tools to generate a list of 291 potential SNPs, 80 of which were included in a barcoding toolkit that is easily implemented using MassARRAY mass spectrometry technology. As a proof‐of‐concept, we used the genotyping toolkit to infer potential hybridization between North American white oaks transplanted outside of their native range (*Q. michauxii*,* Q. montana*,* Q muehlenbergii*/*Q. prinoides,* and *Q. stellata*) into a horticultural collection surrounded by natural forests of locally native trees (*Q. alba* and *Q. macrocarpa*) in the living collection at The Morton Arboretum (Lisle, IL, USA). Phylogenetic and clustering analyses suggested low rates of hybridization between cultivated and native species, with the exception of one *Q. michauxii* mother tree, the acorns of which exhibited high admixture from either *Q. alba* or *Q. stellata* and *Q. macrocarpa*, and a hybrid between *Q. stellata* that appears to have backcrossed almost exclusively to *Q. alba*. Together, RAD‐seq and MassARRAY technologies allow for efficient development and implementation of a multispecies barcode for one of the more challenging forest tree genera.

## 1 INTRODUCTION

Oaks (*Quercus*, Fagaceae) are keystone species in forest and savanna ecosystems across the Northern Hemisphere (Cavender‐Bares, 2016). Hybridization in the genus is common and known to be dependent on spatial distribution of parent trees, pollen dispersal and pollination time, and sexual barriers among other factors (Lagache, Klein, Guichoux, & Petit, 2013; Petit, Bodénès, Ducousso, Roussel, & Kremer, 2004). Oak species with similar reproductive strategies and overlapping geographic regions often hybridize in natural stands (Chybicki & Burczyk, 2010; Curtu, Gailing, & Finkeldey, 2007; Dodd & Afzal‐Rafii, 2004; Dumolin‐Lapègue, Démesure, Fineschi, Le Corre, & Petit, 1997; Dumolin‐Lapègue, Kremer, & Petit, 1999; Efrain Tovar‐Sanchez, 2004; Gerber et al., 2014; Hipp & Weber, 2008; Lexer, Kremer, & Petit, 2006; Moran, Willis, & Clark, 2012; Petit, 1993; Petit et al., 2004; Whittemore & Schaal, 1991). Morphological intermediacy, however, is an imperfect predictor of genetic admixture in oaks, making hybrids difficult to identify (Burgarella et al., 2009; Song, Deng, Hipp, & Li, 2015; Wei, Li, Zhang, & Liao, 2015). Moreover, multispecies hybrid zones are common in oaks and particularly difficult to study due to sampling issues and selection of adequate numbers of informative loci (Craft & Ashley, 2006; Dodd & Afzal‐Rafii, 2004; Peñaloza‐Ramírez et al., 2010; Sullivan, Owusu, Weber, Hipp, & Gailing, 2016).

While hybridization may be a creative force generating new species (Hegarty et al., 2008; Jiggins, Salazar, Linares, & Mavarez, 2008; Mallet, 2007; Meng & Kubatko, 2009; Schumer, Rosenthal, & Andolfatto, 2014), introgressive hybridization is often thought of as a biodiversity‐eroding process, reducing divergence between populations and potentially between closely related species (Lagache et al., 2013; Seehausen, Takimoto, Roy, & Jokela, 2008; Unger, Vendramin, & Robledo‐Arnuncio, 2014; Zaya, Leicht‐Young, Pavlovic, Feldheim, & Ashley, 2015). This is of concern for species conservation, especially as hybridization is expected to increase with anthropogenic changes in climate, environmental homogeneity, and species ranges (Hasselman et al., 2014; Hoban, Mccleary, Schlarbaum, Anagnostakis, & Romero‐Severson, 2012). Living collections in particular, such as botanical gardens and arboreta, introduce large numbers of species to areas outside their native ranges and create potential hybridization zones where species that have long (or always) been allopatric may suddenly be able to exchange genes (Hulme, 2015; Larkin, 2012; Reichard & White, 2001). The degree to which introduced plants within gardens actually do hybridize with local native species, however, is not well known.

In the past, markers such as restriction fragment length polymorphisms (RFLP), amplified fragment length polymorphisms (ALFP), and simple‐sequence repeats (SSR) have been used for population genetics, phylogenetics, and genotype/phenotype studies in a wide range of forest trees, including oaks (Chagné, Lalanne, Madur, Kumar, & Frigério, 2002; Curtu et al., 2007; Durand et al., 2010; Hipp & Weber, 2008; Hipp et al., 2014; Van Droogenbroeck et al., 2004). However, ascertainment bias (Putman & Carbone, 2014), reproducibility issues, and species specificity (Twyford & Ennos, 2012) often limit their utility across a range of species. Moreover, diagnosis of hybrids often requires a high number of loci. EST‐linked SSRs (Durand et al., 2010; Sullivan et al., 2016) and SSRs not known to be associated with coding regions (Craft & Ashley, 2006) have proven valuable for understanding hybridization in systems with a relatively small number of species, but the large number of loci needed to diagnose hybrids in multispecies hybrid zones makes SSRs a difficult marker to apply. With the advent of inexpensive high‐throughput sequencing, SNPs have become a marker of choice for rapid genotyping (Alberto et al., 2013; Chancerel et al., 2011; Lijavetzky, Cabezas, Ibáñez, Rodríguez, & Martínez‐Zapater, 2007; Novaes et al., 2008).

There are several ways to identify 100s or 1,000s of SNPs in model organisms (Appleby, Edwards, & Batley, 2009; Yan et al., 2010). In the past few years, restrictionsite‐associated DNA sequencing (RAD‐seq) and related methods (e.g., genotyping by sequencing, double‐digest RAD) have emerged as particularly efficient approaches to genotype as well as to identify SNPs for further study (Baird et al., 2008; Barchi et al., 2011; Davey & Blaxter, 2010; Pegadaraju, Nipper, Hulke, Qi, & Schultz, 2013; Scaglione et al., 2012). These methods have been applied with great success to understanding phylogeny, species circumscription, and hybridization patterns in oaks (Cavender‐Bares et al., 2015; Eaton, Hipp, González‐Rodríguez, & Cavender‐Bares, 2015; Fitz‐Gibbon, Hipp, Pham, Manos, & Sork, 2017; Hipp et al., 2014; McVay, Hauser, Hipp, & Manos, 2017). However, the costs are often relatively high per individual and require a library of identified individuals for comparison, making RAD‐seq and related methods impractical for identification of individual trees outside of the context of tree diversity research programs.

DNA barcoding is often problematic in trees due to lineage sorting, hybridization, or low polymorphism (Arca et al., 2012; Hollingsworth, Graham, & Little, 2011; Percy et al., 2014; Simeone, Piredda, Papini, Vessella, & Schirone, 2013). Oaks are no exception in this regard: to date, an oak DNA barcode has seemed impracticable, as no combination of a small number of loci provides reliable species diagnosis (Denk & Grimm, 2010; Hubert et al., 2014; Hipp, 2015; Oh & Manos, 2008; Piredda, Simeone, Attimonelli, Bellarosa, & Schirone, 2011; Simeone et al., 2013; though cf. Chen, Zeng, Liao, Yan, & Zhang, 2017 and Yang et al., 2017 for recent successes in East Asian oaks). In this study, we present an 80‐SNP DNA barcode for species identification and hybrid studies of a suite of common eastern North American white oaks. Our addresses solves the problem of identifying barcode‐informative loci through careful selection of a core set of informative and easily amplified SNPs from a broadly sampled RAD‐seq dataset (McVay, Hipp, & Manos, 2017). We then develop PCR primers for multiplex amplification and SNP genotyping using the MassARRAY platform and apply this DNA barcode to a pilot study of hybridization among white oaks in the living collections of The Morton Arboretum. The resulting genotyping toolkit builds on a previous 2‐species oak barcode using MassARRAY technology (Guichoux, Lagache, Wagner, Léger, & Petit, 2011) and provides a model for rapid, inexpensive, high‐throughput assessments of hybridization and species identity in multispecies oak stands and living collections.

## 2 MATERIALS AND METHODS

### 2.1 Sampling

**Figure 1:**
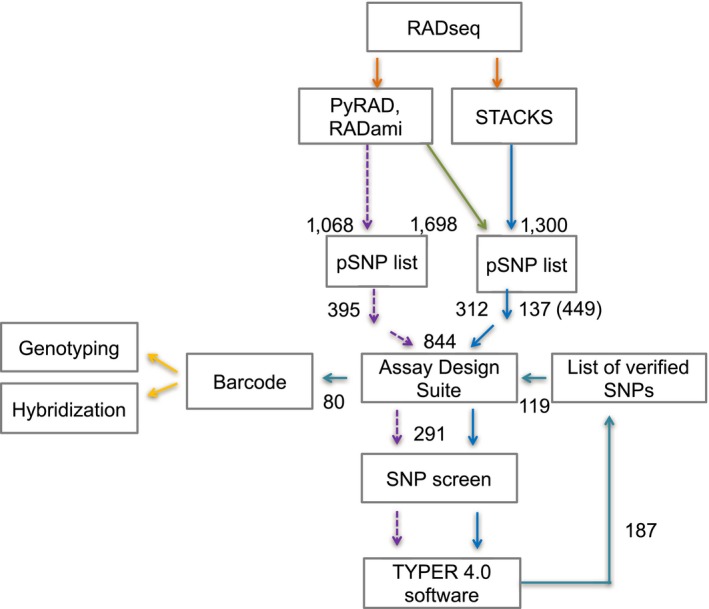
Flow diagram outlining analysis used in this study and future directions. RAD‐seq (orange arrows) data from previous project were analyzed using PyRAD/RADami and *Stacks*. A stringent approach (dotted purple arrows) and relaxed approach (solid blue arrows) were used to develop two lists of potential SNP (pSNPs). These were subjected to the primer design software Assay Design Suite and data analysis using TYPER 4.0 software, screened, and checked for SNP validation. A list of verified SNPs was re‐entered to the primer design software to select for 80 suitable SNP multiplexes, which comprise the barcode (turquoise arrows). Applications of the barcode include genotyping of North American white oaks and hybridization studies (yellow arrows)

For marker development, we used RAD‐seq data from 63 samples selected to represent 15 species or species groups (Supplementary File 1) reported from a previous study (McVay, Hipp et al., 2017). *Quercus muehlenbergii* and *Q. prinoides* were treated together as a single species in our study, because our analyses as well as current phylogenetic work (McVay, Hipp et al., 2017; Pham, Hipp, Manos, & Cronn, 2017) suggest that the two are not distinguishable using multilocus nuclear data. The European white oaks and East Asian white oaks were treated as two species groups to identify markers that distinguish the two groups from one another and to study possible patterns of gene flow between American and Eurasian white oak. We did not, however, attempt to distinguish the Eurasian white oak species from one another, as our focus is on hybridization among eastern North American species. For SNP evaluation (Figure 1), we included 40 of the 63 trees used for SNP identification and an additional 55 trees from 16 species (Supplementary File 1). Of the pool of 95 samples used for SNP screening, 59 trees are designated as learning samples in our study, as their sequences were used to develop the SNPs utilized in this study (Supplementary File 1).

**Figure 2:**
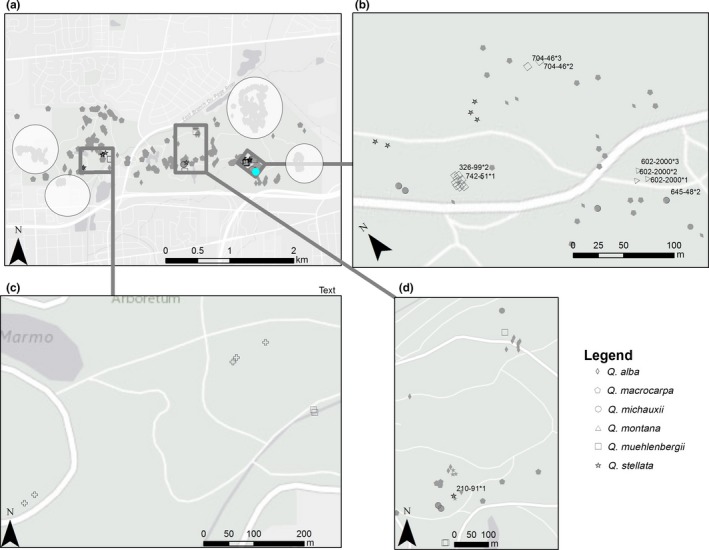
Fine‐scale map of the study site in the oak living collections at The Morton Arboretum. (a) Overview map of the mother and potential father trees genotyped in this study. Species are indicated with shapes (see legend). Mother trees are represented with black‐colored shapes. Distribution of nearby trees not genotyped is indicated with gray‐colored shapes. Potential fathers are indicated with white‐colored shapes. (b) Selection of eight mother trees. (c) Selection of potential father trees and nearby trees. (d) Selection of *Q. stellata* mother trees, potential fathers, and nearby trees. Legend: *Q. stellata* (cross), *Q. alba* (diamond), *Q. michauxii* (circle), *Q. macrocaropa* (pentagon), *Q. muehlenbergii* (square), *Q. montana* (triangle), *Q. prinoides* (arrow)

A map displaying the locations of Arboretum cultivated trees, studied plants, and nearby *Quercus* spp. was produced in ESRI ArcMap 10.3.1 (Figure 2). Existing feature classes containing the GPS coordinates of plants in The Morton Arboretum's Living Collections were utilized and displayed over a basemap provided by OpenStreet Maps and the legend was edited in Paint.NET (http://www.getpaint.net ).

### 2.2 RAD‐seq genotyping and clustering

RAD‐seq DNA extraction, library preparation, and sequencing were conducted as presented previously in oaks (Cavender‐Bares et al., 2015; Hipp et al., 2014, 2018; McVay, Hauser et al., 2017, McVay, Hipp et al., 2017). Briefly, DNA for all RAD‐seq samples was extracted from fresh or frozen material using the DNeasy plant extraction protocol (DNeasy, Qiagen, Valencia, CA, USA). DNA extractions were gel‐quantified in agarose by visual comparison with a 1 kb+ Ladder (Invitrogen, Thermo Fisher Scientific, Waltham, MA, USA). Extraction concentrations ranged from 5 to 10 ng DNA/μl extraction. RAD sequencing library preparation was conducted at Floragenex, Inc. (Portland, OR, USA) following the methods of Baird et al. (2008) with *Pst*I as the restriction enzyme. RAD libraries were barcoded by individual and multiplexed on an Illumina Genome Analyzer IIx or on Illumina HiSeq 2000. Sequencing reads were 100 bp in length; after removal of the barcode, analyzed sequences were 85 bp long. Quality, read lengths, and base composition of FASTQ data were assessed in R v. 3.3.1 (“Bug in your hair”; R Core Team 2016) using the ShortRead package (Morgan et al., 2009). FASTQ files are publically available under NCBI BioProject PRJNA376740 (https://www.ncbi.nlm.nih.gov/bioproject/376740).

RAD‐seq fastq data were analyzed using PyRAD version 1.64 (Eaton, 2014) (http://www.dereneaton.com/software). In this pipeline, sequences are clustered first by individual using USEARCH (Edgar, 2010), which allows sequences within clusters to vary in indels, nucleotide polymorphisms, and sequencing strand (direction). After clustering, heterozygosity and sequencing errors are jointly estimated from the base counts observed across all sequences, sites, and clusters using the likelihood equation of Lynch (2008). Heterozygotes are inferred by a binomial probability based on these parameters. Bases that could not be assigned with ≥95% probability were treated as unknown (*N*). Any locus possessing more than two haplotypes within individuals after correcting for sequencing errors was discarded, under the assumption that it included one or more paralogous sequences. For each individual, each locus was summarized as a consensus sequence, and consensus sequences were clustered among individuals to generate a data matrix for each locus. Due to variation in sequencing coverage and mutation at the restriction site defining RAD loci, the resulting data matrix was not complete for all loci in all individuals. The following parameters were specified for PyRAD: minimum depth of reads per within‐sample cluster: 10; maximum number of sites in a read that can have a quality score of less than twenty: 4; clustering threshold (percent similarity): 0.85; minimum number of samples in each across‐sample cluster: 20; maximum number of individuals with a shared heterozygous site in an across‐sample cluster: 3. All other settings used default values.

For comparison, RAD‐seq data were also clustered using *Stacks* (Catchen, Amores, Hohenlohe, Cresko, & Postlethwait, 2011; Catchen, Hohenlohe, Bassham, Amores, & Cresko, 2013). Barcodes were first removed using *gawk* prior to filtering out low‐quality reads with the “process_radtags” module of *Stacks* (Catchen et al., 2011, 2013). The wrapper program “denovo_map.pl” provided by *Stacks* was used to perform de novo assembly of RAD‐seq. Ten reads were set as the minimum to be considered as a stack (m); distance between two stacks to be assembled to loci was set to two (M); and the distance between loci to be merged was set to four (*n*). Filtering criteria were set to 75% of individuals in a population required to be present in a loci (*r*) and 10 as the minimum number of populations in which a locus must be present to be considered (*p*).

### 2.3 SNP identification in RAD‐seq dataset

SNPs were identified using two methods. In the first, pairwise F_ST_ was calculated between all pairs of oak species using the RADami (version ≥1.1‐2) (Hipp et al., 2014) and hierfstat (version 0.04‐10) (Goudet, 2013) packages of R. The resulting F_ST_ matrix identified loci with fixed or nearly fixed differences (pairwise *F*_ST_ ≥  0.95) between any pair of white oak species, identified the location of SNPs within the locus, and identified how many pairwise comparisons were at or above the threshold *F*_ST_ level (of 0.95). This approach generated a list of more than 1,090 loci containing 4,020 potential SNPs of *F*_ST_ ≥ 0.95, all of which were exported as FASTA files. Loci were then filtered under both stringent criteria and relaxed criteria. The stringent approach excluded SNPs with ambiguity codes of any kind and resulted in 1,068 potential SNPs (Supplementary File 2). These were further subjected to manual inspection, and SNPs were retained if they were present in at least 50% of individuals from one species to be considered for SNP screening; this threshold was chosen to maximize the number of SNPs we could consider in design without wasting undue time on SNPs that might provide little power to distinguish among species. FASTA files were formatted with custom bash scripts that retrieved one species‐specific SNP per locus sequence and masked other SNPs using IUPAC ambiguity codes (Supplementary File 2). The relaxed approach treated heterozygotic and ambiguous positions as potentially informative, as long as at least one individual per species was homozygous at the position distinguishing species; this expanded the list of potential SNPs to 1698. For both the relaxed and stringent approaches, loci were considered suitable for SNP identification only if they had at least 20 bp available for primer design at both the 5′ and 3′ ends.

For *Stacks* data, the program “populations” was used to compute pairwise F_ST_ between species. Loci were derived from the vcf output file. Output files (vcf and fasta) were obtained and further processed with custom bash scripts and manually selected in Excel (Microsoft, Washington State, USA) to create a list of potential SNPs. The corresponding position of a SNP within a RAD‐seq stack was identified by a custom script from the output files (sumstats and vcf; Supplementary File 3).

Stand‐alone NCBI‐BLASTN version 2.2.30 (Altschul, Gish, Miller, Myers, & Lipman, 1990) was utilized to determine overlap of loci between PyRAD and *Stacks*. The command “makeblastdb” was used to convert each loci dataset into a database. BLASTN (version 2.2.30+) was used to compare PyRAD loci to a *Stacks* loci database followed by reciprocal BLASTN of *Stacks* loci against a PyRAD loci database. The e‐value of 1E−05 was selected as the threshold for recognizing loci as orthologous; this is a commonly used value and is relatively arbitrary, but given the restricted nature of the dataset provides a framework for comparing loci. The output was saved as tabular format. Loci that matched between the PyRAD and *Stacks* outputs with alignment length exceeding more than 95% were treated as duplicates and excluded from downstream analyses.

### 2.4 Primer design

Assay Design 4.0 Suite (Agena Biosciences, San Diego, CA, USA) was used to design primers for MassARRAY analysis (Bradić, Costa, & Chelo, 2012). A maximum of 40 SNPs were multiplexed, and a total of four multiplexes were screened per chip. Primers with a high percentage of Ns, indels, and primer‐dimer formation potential were not considered for multiplexing.

### 2.5 SNP genotyping

SNP screening and genotyping were performed using TYPER 4.0.20 software by Agena Biosciences. Spectrum display of the mass and intensity coordinates for homozygous and heterozygous SNPs was manually corrected for unclear genotyped samples, and only clear calls were retained. The results were exported as xml files and visually inspected in Microsoft Excel.

### 2.6 SNP evaluation

After SNP screening, SNPs present in all individuals of a species were marked as fixed SNPs, whereas those present in the majority of individuals were marked as nearly fixed; 11.6 ± 7.7 (*SD*) SNPs selected as diagnostic for each of the 13 species and two species groups targeted (Table 2) were in the SEQUENOM‐verified set of 187 SNPs. Of the species sampled, only *Q. boyntonii* failed to yield any verified SNPs: All other species yielded four or more. Matrices of 40, 80, and 119 verified SNPs from the 187 screened SNPs were created with an average of five to six SNPs to distinguish among the species *Q. alba*,* Q. bicolor*,* Q. lyrata*,* Q. michauxii*,* Q. muehlenbergii/prinoides*,* Q. montana,* and *Q. stellata* (Table 2, “SNPS verified”). The matrix of 119 SNPs was chosen for design of two final multiplexes to ensure a selection of 80 SNPs via the primer design suite across two multiplexes. Maximum‐likelihood (ML) phylogenetic analysis was then conducted for each matrix to evaluate whether each SNP set could identify species known from prior work (Hipp et al., 2018; McVay, Hipp et al., 2017) to be monophyletic. Phylogenetic analysis was conducted using PhyML (Guindon et al., 2010) with mode = “A la carte” and number of bootstraps = 100, as implemented in phylogeny.fr (Dereeper et al., 2008). FigTree version 1.4.2 (http://tree.bio.ed.ac.uk/software/figtree/) was used to visualize results.

### 2.7 Sampling and DNA extraction from white oak acorns

Acorns were collected in fall of 2013 and 2014 from nine mother trees in The Morton Arboretum oak collection site (Figure 2). Mother trees were selected to be species of Illinois that are not native to The Morton Arboretum. Three species—*Quercus stellata*, the subject of a detailed distribution study (Prasad, Gardiner, Iverson, Matthews, & Peters, 2013), *Q. michauxii*, and *Q. montana*—are southern species that have the potential to migrate north with climate change. The fourth, *Q. muehlenbergii*/*Q. prinoides*, ranges to northern Illinois but is not present in native stands on the grounds of The Morton Arboretum. Acorns were subjected to a five‐minute floating test in cold water. Any floating acorns were discarded. The remaining acorns were counted and underwent cold stratification for 2 months. A maximum of 50 acorns per species were planted and grown in the glasshouse for 3 months, with the exception of *Q. montana* with 100 acorns. After germination, leaves were harvested and stored in zipper locking bags with paper towels to remove excess moisture and stored at −4°C.

Leaf tissue (1 cm^2^ per tree) was placed in 96‐well plates and stored in a −20°C freezer. Prior to DNA extraction, plates were placed overnight into a vacuum desiccator. Dried leaves were ground using metallic beads for 1 min (twice) at 1,500 rpm (estimated 24‐‐28 G’s; S. Geiger, SPEX SamplePrep LLC, pers.comm.) on a Geno Grinder (SPEX SamplePrep, Metuchen, NJ, USA). DNA was isolated using Plant Prep Adem‐Kit (Ademtech, Pessac, France) according to manufacturer's instructions. DNA concentrations were estimated using Nanodrop spectrophotometer (NanoDrop Technologies, Wilmington, DE, USA), and concentrations were set to 10 ng/μl using STARlet 8‐channel robot (Hamilton, USA).

### 2.8 Postgenotyping analysis

Following MassARRAY genotyping, SNPs were concatenated and converted to Phylip format for phylogenetic analysis. UPGMA trees were calculated in Geneious R9 (Kearse et al., 2012). The RAxML BlackBox Web server was used to run ML analysis with default settings (Stamatakis, Hoover, & Rougemont, 2008), and trees were uploaded to iTOL for annotation (Letunic & Bork, 2016). The Eurasian white oaks (*Q. petraea*,* Q. robur*,* Q. fabri*, and *Q. aliena*) were used to root the ML tree for purposes of visualization. Dataset templates retrieved from the iTOL help page were used for color‐coding species (with the exception of the Eurasian white oaks, which were colored the same) and to highlight mother trees and learning samples (Supplementary File 4).

**Figure 3:**
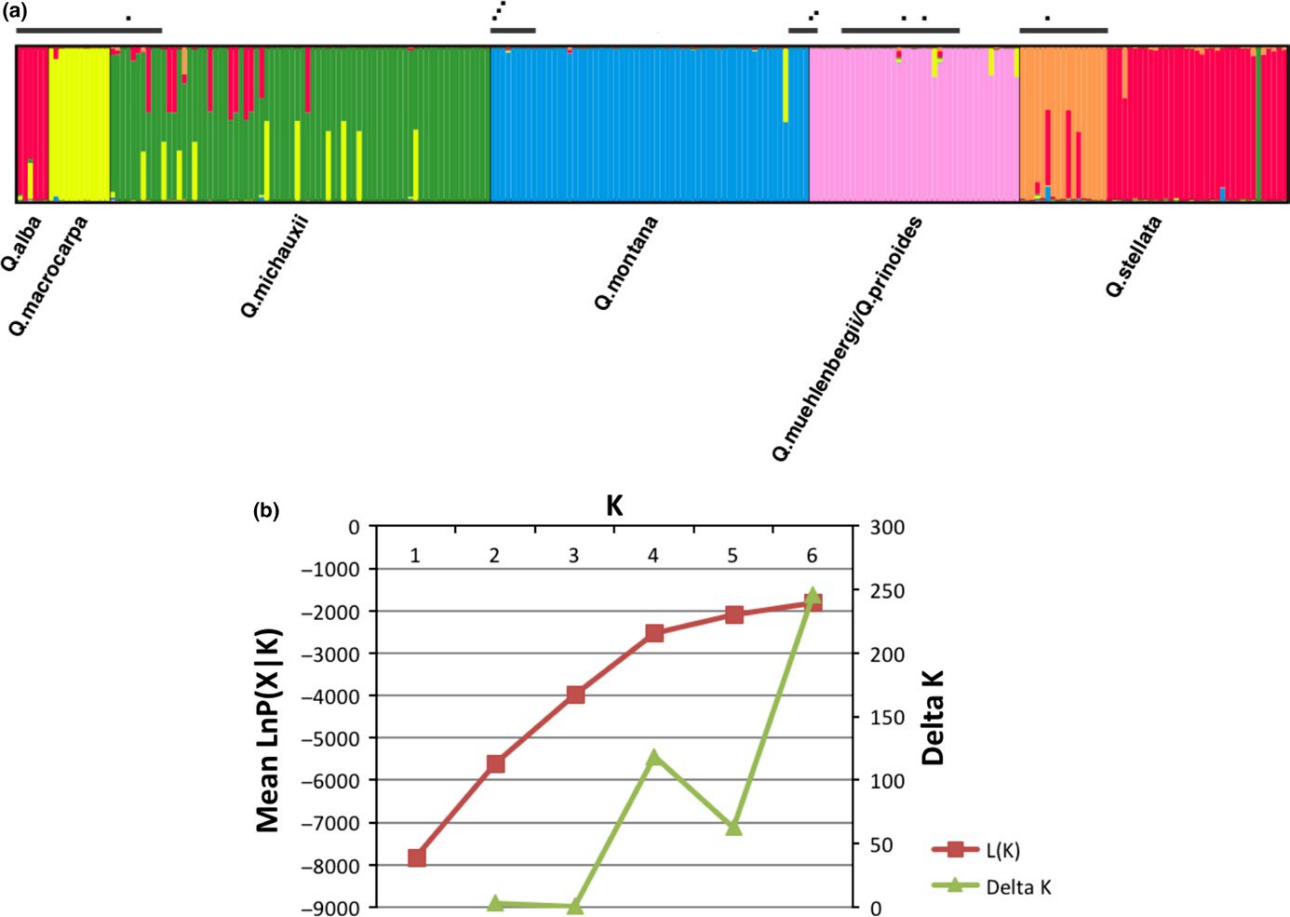
STRUCTURE analysis to assess rate of hybrid offspring by non‐native North American white oaks within the oak living collections at The Morton Arboretum. (a) Genetic assignment of 247 individuals at 66 loci according to MCMC clustering method implemented in STRUCTURE (Pritchard et al., 2000). DISTRUCT v1.1 was used for the visualization (Rosenberg, 2004). Vertical black lines represent grouping of individuals according to their population assignment. Each vertical line color‐codes the genotype probability of an individual according to population grouping. Black bars over individuals represent adult trees. Black dots over individuals represent mother trees producing acorns genotyped in this study. (b) Estimation of best cluster number (*K*) calculated by STRUCTURE with respect to mean log likelihood of the data over five iterations (left axis) and ΔK plotted as a function of *K* (*K* = 1–6) (right axis)

Admixture in acorns from mother trees was investigated using an admixed population genetic model as implemented in STRUCTURE v. 2.3.4 (Pritchard, Stephens, & Donnelly, 2000). For every sample, SNP alleles were coded as integers (A = 1, C = 2, T = 3, G = 4, and missing data = −1), and the species of each sample was coded in the population column. Analysis was conducted under the “admixture” ancestral model with allele frequencies correlated among populations and lambda (λ) set to the default value (1.0). Population identity was not used as a prior. Markov chain Monte Carlo (MCMC) was run using a burn‐in period of 100,000 MCMC steps followed by 1,000,000 data‐gathering steps. Markov chains were run from *K *= one to six populations, five replicates for each value of *K*. To estimate the best number of clusters (*K*) given the data, mean Δ*K* over replicates was calculated (Evanno, Regnaut, & Goudet, 2005; Owusu, Sullivan, Weber, Hipp, & Gailing, 2015). *K *=* *6 showed the highest support based on Δ*K* (Figure 3b).

## 3 RESULTS

Using the methods described above, we worked from a set of 32,959 RAD‐seq loci to a candidate set of 844 SNPs. We developed primers for 344 loci (Table 1) based on inspection of the candidate set, of which 291 were screened using the MassARRAY system; 187 SNPs amplified successfully, and 119 were selected for multiplexing using the MassARRAY primer design tool (Figure 1). From this, we developed a final DNA barcode for 15 oak species/species groups based on 80 SNPs, which can be run in two MassARRAY multiplexes. We then used this DNA barcode to validate identity of the 59 learning samples used to develop the barcode and successfully evaluate species status of 37 previously nongenotyped adults and 228 acorns from nine mother trees in an Arboretum setting.

**Table 1:** Assay design suite results

	Total	Passed	Multiplexed
First SNP screen	395	131	131
Second SNP screen	449	213	160
Both screens	844	344	291

Detailed breakdown of the SNP screens. Total corresponds to the established lists of potential SNPs and was considered for the primer development. “Passed” represents the number of SNPs meeting the criteria for primer development. “Multiplexed” represents the number of SNPs successfully multiplexed with the maximum of 40 SNP per multiplex and ordered for screening.

### 3.1 SNP identification in RAD‐seq data

Two different approaches were used to generate a list of potential SNPs for barcoding. First, the stringent screening approach resulted in the identification of 1090 loci of potential utility, which were exported as FASTA files (Figure 1, dashed line). We generated a list of 395 potential SNPs of which the majority (264 SNPs) were discarded by the primer design tool and 131 showed primer compatibility across four multiplexes. Of the 131 SNPs, 83 (63%) were added to the list of verified SNPs. The majority of these SNPs were diagnostic for *Q. bicolor*,* Q. michauxii*, or *Q. lyrata*. For *Q. michauxii,* we found more than fixed SNPs that were considered for the genotyping toolkit.

The relaxed screening approach (Figure 1, green line) was then used primarily to identify SNPs for *Q. alba*,* Q. macrocarpa*,* Q. montana*,* Q. muehlenbergii*/*Q. prinoides*,* Q. stellata* and representatives of the Eurasian and East Asian white oaks. This second screening approach was supplemented with *Stacks*‐identified loci (Figure 1, blue line). Here, we identified 1300 potential SNPs of which 137 were fixed or nearly fixed in *Q. alba*,* Q. muehlenbergii/prinoides*,* Q. stellata*,* Q. macrocarpa*, and *Q. montana* with at least 20 bp available at both ends for primer development. Combined, the relaxed approach of PyRAD, RADami, and *Stacks* resulted in 449 potential SNPs that were considered for primer development (Table 1). Of these, 312 were present in *Q. alba*,* Q. macrocarpa*,* Q. montana*,* Q. muehlenbergii*/*Q. prinoides*,* Q. stellata*. It is worth noting that the total number of SNPs is strongly constrained by the need for a 20‐bp region at both ends of the RAD‐seq fragment for primer development: This constraint alone decreased our initial candidate list of SNPs from 1698 to the final 449 that we could subject to primer design. Of 1090 loci from PyRAD and 1300 loci from *Stacks*, only 24 loci overlapped, and these duplicates were removed.

### 3.2 Primer design and SNP genotyping

The Assay Design Suite was used to design suitable primers pairs for more than 844 potential SNPs, 395 from the stringent selection approach and 449 from the relaxed selection approach. The primer design software rejected 59% of these due to the presence of ambiguous nucleotides close to the SNP site, potential primer‐dimer formation, or placement of SNP sites too close to the 5′ or 3′ end of the amplified region. Primers were designed for the remaining 344 (40%) potential SNPs, of which we combined 291 SNPs into eight multiplexes for screening (Table 1); 187 of these SNPs were confirmed as fixed (44) or nearly fixed (143) in a species, while 93 SNPs were identified as false positives based on failure to discriminate species in a broader range of 63 samples. Eleven SNPs did not amplify. Among 44 fixed SNPs, the majority belonged to *Q. michauxii* (11) followed by *Q. muehlenbergii/prinoides* (6) (Table 2). *Quercus montana* exhibited the most nearly fixed SNPs (13). The majority of *Q. stellata* SNPs (15) were found to be heterozygous or overlapping with another species. The most false positives were determined for *Q. muehlenbergii/Q. prinoides* and representatives of the Eurasian white oaks. This is somewhat remarkable given that *Q. muehlebergii*/*prinoides* was represented by six individuals in the initial RAD‐seq screening, comparable to the number we had for *Q. alba* (4) and *Q. michauxii* (5).

**Table 2:** Success rate of SNPs

Species	*N*	SNPs start	SNPs design	SNPs verified	SNPs barcode	SNPs STRUCTURE
*Q. alba*	4	95	30	20	5	3
*Q. austrina*	2	25	8	8	5	4
*Q. bicolor*	2	22	10	7	5	4
*Q. boyntonii*	2	2	1	0	0	0
*Q. chapmanii*	2	10	4	4	1	1
*Q. lyrata*	4	35	8	7	4	3
*Q. macrocarpa*	9	64	15	7	4	3
*Q. michauxii*	5	57	23	19	8	5
*Q. montana*	4	84	33	25	8	7
*Q. muehlenbergii/Q. prinoides*	6	143	45	25	8	7
*Q. oglethorpensis*	3	22	7	6	4	4
*Q. sinuata*	2	31	16	13	8	7
*Q. stellata*	3	52	26	21	13	12
Clade 1	14	7	6	7	2	1
Clade 2	4	3	0	0	0	0
East Asian white oaks	4	98	29	10	4	4
European white oaks	11	94	30	8	1	1
Total		844	291	187	80	66
Mean (± *SD*)		49.6 (±41.1)	17.1 (±13.2)	11 (±8.1)	4.7 (±3.5)	3.9 (±3.1)

Initial number of SNPs (RADami and *Stacks*), number of SNPs after passing primer design software with percentage in parentheses, and number of SNPs verified by MassARRAY. Clade 1 = *Q. sinuata*,* Q. oglethorpensis*,* Q. boyntonii*,* Q. stellata*,* Q. chapmanii*,* Q. austirna*. Clade 2 = *Q. austrina* and *Q. chapmanii*. **SNPs start** = number of SNPs initially selected for consideration. **SNPs design** = SNPs that passed assay design step. **SNPs verified** = SNPs that successfully amplified on SEQUENOM. **SNPs barcode** = SNPs included in the two‐multiplex barcoding set presented here. **SNPs STRUCTURE** = SNPs included in STRUCTURE analyses presented in this article (removing SNPs with substantial amounts of missing data).

The remaining 187 (65%) of the screened SNPs showed the dinucleotide pattern indicated during SNP identification using the RAD‐seq data and were maintained in this study. We reduced the list of verified 187 SNPs to 119 by selection for a minimum of five to six SNPs per species (Figure 1). Of 119 potential SNPs, 80 were selected and distributed into two multiplexes by primer design software and used to test for hybridization of collected and germinated acorns (Supplementary File 5). Only 70 amplified reliably for our samples and are presented in analyses below; primers for all 80 SNPs constituting the full oak DNA barcode are presented in the supplement.

### 3.3 Analysis of SNP data

**Figure 4:**
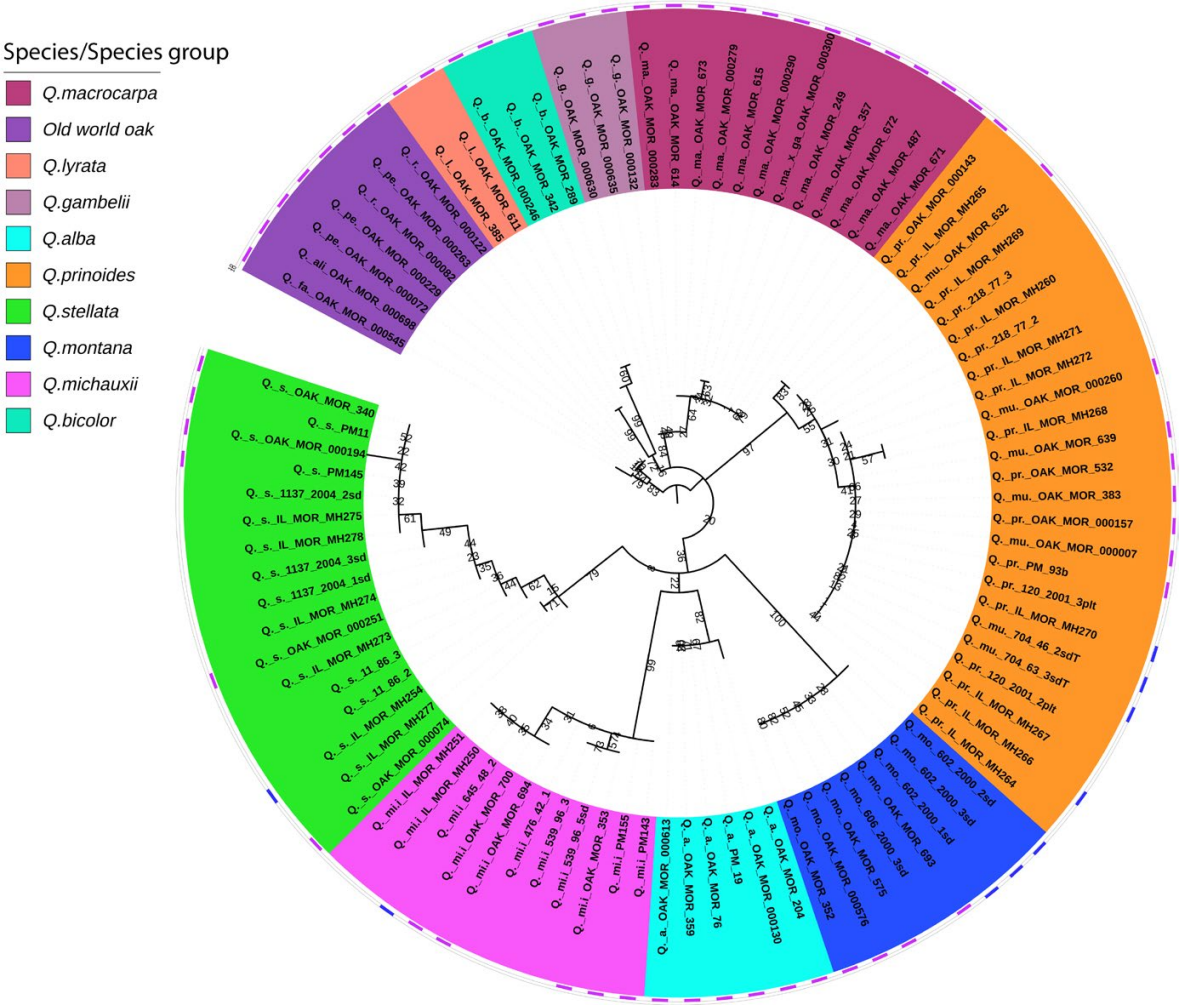
Maximum‐likelihood tree of mother trees, potential father trees, and learning samples. Species are color‐coded (see legend). The mother trees genotyped in this study are marked with blue boxes. Learning samples are indicated with purple boxes. *Q. aliena*,* Q. fabri*,* Q. robur,* and *Q. petraea* are combined in this study and represented as Eurasian (Old World) white oaks. The tree is rooted by the Eurasian white oaks. Annotations were made in iTOL (Letunic & Bork, 2016)

The developed barcoding toolkit was used to genotype a total of 324 samples. The sample set consisted of 228 acorns, 37 adult trees not previously genotyped (including nine mother trees), and 59 learning samples drawn from the 63 samples originally sequenced using RAD‐seq and used to identify SNPs. Maximum‐likelihood analysis of the SNP dataset from parent trees and learning samples recovers a topology comparable to that described in previous studies (Hipp et al., 2014, 2018; McVay, Hipp et al., 2017) (Figure 4). UPGMA analyses provide no additional insights over maximum‐likelihood analyses and are excluded from further discussion.

A total of 70 SNPs in two multiplexes were successfully amplified. Both multiplexes together were required to identify species. Amplification of SNPs varied among samples. The MassARRAY amplification success rate per mother tree was assessed by calculating the percentage of missing data per seedling for the two multiplexes and summarized by mother tree (Table 3). Overall, the rate of MassARRAY amplification is high, ranging from 57% to 100%, with the exception for two mother trees, *Q. montana* (602‐2000*3) and *Q. muehlenbergii* (704‐46*2 sd T) (Table 3).

**Table 3:** Germination and MassARRAY success rate

Individual ID	Species	*N*	Germinated	Amplification rate		Hybridization frequency (%)
				100%–71%	70%–0%	
645‐48*2	*Q. michauxii*	100	70 (70%)	63 (90%)	7	34.9
602‐2000*1 sd	*Q. montana*	50	45 (90%)	32 (71%)	13	3.1
210‐91*1	*Q. stellata*	71	43 (60%)	35 (81%)	8	5.71
602‐2000*2	*Q. montana*	50	35 (70%)	20 (57%)	15	0
704‐46*2 sd T	*Q. muehlenbergii*	50	12 (24%)	3 (25%)	9	0
326‐99*2	*Q. prinoides*	29	9 (31%)	6 (66%)	3	0
742‐51*1	*Q. prinoides*	67	8 (12%)	6 (75%)	2	33.3
602‐2000*3	*Q. montana*	7	5 (70%)	2 (40%)	3	50
704‐46*3 sd T	*Q. muehlenbergii*	6	1 (16%)	1 (100%)	0	0

Listed are the individual IDs and species names of the corresponding nine mother trees. The amount of collected and planted acorns in Fall/Winter of 2014 per mother tree and their germination rate. The table is sorted by the number of germinated acorns. The amplification rate is divided into two categories: 71%–100% of markers amplified and 0%–70% of markers amplified. Hybridization frequency per mother tree is based only on individuals for whom 71%–100% of markers amplified.

Species were recovered as monophyletic based on the barcode SNP set with bootstraps values between 62% and 100% (Figure 4). During SNP screening based on adult trees only, we detected two potential hybrids: *Q. macrocarpa*‐616 (OAK‐MOR‐000616) and *Q. stellata*‐74 (OAK‐MOR‐000074). Morphologically, the former sample also had the appearance of being intermediate between *Q. macrocarpa* and *Q. stellata*. Phylogenetically, *Q. macrocarpa*‐616 was placed either outside of *Q. oglethorpensis* or wedged between clade 1 (*Q. sinuata*,* Q. oglethorpensis*,* Q. boyntonii*,* Q. stellata*,* Q. chapmanii*,* Q. austrina*) and clade 2 (*Q. austrina* and *Q. chapmanii* and *Q. macrocarpa*/*Q. gambelii)*. *Quercus stellata*‐74 was placed next to the clade of *Q. alba,* suggesting hybrid origin (Supplementary File 4).

### 3.4 Species integrity in a botanical garden

We used the barcoding toolkit to genotype nine mother trees from five species not native to the region and 228 acorns from them, as well as 28 additional adult trees (Table 3). STRUCTURE analysis was performed with 247 samples using 66 loci to estimate rates of hybridization in the genotyped samples. A *K* of 6 explained substantial variance in the data without overfitting (Figure 3b). Of 228 acorns, 191 acorns could be assigned to a single species, whereas 24 acorns showed >5% admixture (Figure 3). The majority of hybrid acorns were identified from a single mother tree, *Q. michauxii* 645‐48*2. Of 63 acorns from this mother tree, 21 were admixed with either *Q. alba* or *Q. stellata* or *Q. macrocarpa* (Figure 3a). We observed no hybridization among acorns from the *Q. muehlenbergii* mother tree; however, two acorns from *Q. prinoides* 745‐52*1 showed admixture with *Q. macrocarpa*. An interesting result was observed for *Q. stellata*. Here, the adult trees separate from the progeny, but three adults—one of which includes the mother tree of the acorns germinated for this study—genotyped as hybrids with *Q. alba*. The progeny then genotyped as *Q. alba* or a hybrid between *Q. alba* and *Q. stellata* (one plant) rather than *Q. stellata* (Figure 3a). Furthermore, of 35 acorns from *Q. stellata* 210‐91*1, two exhibited admixture from *Q. montana* or *Q. macrocarpa* (Table 3).

One acorn in our study (Quercus_stellata_STE_210_91_1_47) appears to be misclassified as *Q. michauxii* (Figure 3a). As the half‐siblings of this individual genotype as *Q. alba* or as hybrids between *Q. alba* and *Q. stellata*, we treat this individual as a labeling error during leaf harvest or DNA extraction.

## 4 DISCUSSION

Advances in next‐generation sequencing technologies allow researchers to generate high amounts of reduced‐representation data by RAD‐seq, GBS, anchored enrichment, and other methods at relatively low per‐base‐pair cost. Such methods reduce the risk of ascertainment bias, as the primary criteria for developing genotyping strategies using these method are generally associated with marker number rather than variability per se. Moreover, although the choice of the restriction enzymes in RAD‐seq, GBS, and related methods may introduce genome sampling bias (as, for example, methylation‐sensitive sites are more frequent in highly transcribed regions; Heslot, Rutkoski, Poland, Jannink, & Sorrells, 2013), these methods tend to sample broadly across the genome. However, the per‐individual costs of these studies often stand in the way of genotyping the large numbers of individuals needed to investigate species coherence, hybridization, and other population‐level dynamics. In the current study, we started with a broad sample of RAD‐seq data, representing more than 65 oak individuals, generated for a phylogenetic study of the North American white oaks (McVay, Hipp et al., 2017) to identify SNPs that can be inexpensively genotyped for large numbers of individuals. The result is an inexpensive and easy‐to‐use 80‐SNP genotyping toolkit to distinguish the common white oaks of eastern North America. The toolkit has proven effective for an initial screen of hybrids resulting from outcrossing in the cultivated oaks of The Morton Arboretum.

We expect that the approach presented here will be useful for ecological studies involving white oaks of the region. Most of the species used to develop the SNPs are widely distributed across eastern North America, making our toolkit useful for hybridization studies in natural stands. Of broader interest, our study demonstrates that a species‐level oak barcode can be developed in a region where numerous species occur sympatrically, enabling researchers to confirm identifications in seedlings, root specimens, or other material that is not readily identified. Our focus was to develop a genotyping tool specifically for predominant white oaks of the North Americas. Given the difficulties of phylogenetic inference in forest trees and oaks in particular, our targeted barcode is *not* an appropriate tool for community phylogenetics or biodiversity discovery (Kress, García‐Robledo, Uriarte, & Erickson, 2015; Kress et al., 2009) or inferring phylogenetic relationships among large numbers of individuals within diverse clades (The Global Carex Group et al., 2016). It is, however, appropriate to investigations of species identity and hybridization among white oaks of eastern North American deciduous forests.

### 4.1 Comparing pipelines: Stacks vs PyRAD

The bioinformatic queries of the RAD‐seq dataset we undertook used different strategies to identify SNPs of potential genotyping value. PyRAD uses an alignment‐based algorithm (USEARCH/VSEARCH for clustering, MUSCLE for multiple alignment) to detect loci whereas *Stacks* uses an “off‐by‐*N*” threshold for clustering. It has been demonstrated that *Stacks* may recover as few as half the loci PyRAD does when indels are frequent (Eaton, 2014). In our study, the use of PyRAD for locus detection resulted in a higher number of SNPs that we initially screened (233 SNPs) and subsequently verified via MassARRAY and considered for the barcode (100 SNPs). Using *Stacks*, we identified ~25% as many SNPs (58 SNPs screened, 19 suitable for genotyping). PyRAD is expected to outperform *Stacks* in phylogenetic samples (Eaton, 2014), making it a better fit to our multispecies sample. *Stacks* is more suitable for the analysis of population data (Catchen et al., 2013) and may be as effective as PyRAD for within‐species studies or studies among two close relatives, but our results support previous findings that at least in a phylogenetically structured multispecies context, PyRAD is likely to be more efficient at detecting shared loci.

### 4.2 Constraints on primer design

The RAD‐seq loci we used to design primers were approximately 85 bp in length (ignoring indels), which introduces a strong constraint on primer design. The quality of flanking sequences of a SNP contributes strongly to its suitability for multiplexing, although the MassARRAY primer design software does accept ambiguities within the flanking region. Our short loci constrained us to a 60‐bp maximum length region in the middle of each locus where we could amplify SNPs; any SNPs outside this region were not relevant to the study, reducing by roughly 75% the amount of the genome readable in our SNP assay relative to the amount genotyped using RAD‐seq. This limitation could easily be circumvented using longer sequencing reads. In our study, primer design and multiplex suitability cut our list of potential SNPs by half: Of more than 800 potential SNPs, only 187 reached the final set of SNPs that we considered in detail for the final reactions. The high rate of attrition expected during any such project makes increasing sequence read length at the outset of the project—thus increasing the percentage of the genotype sequenced that is suitable for SNP amplification—an efficient way of increasing the number of potential SNPs.

### 4.3 SNP quality

In most traditional genotyping projects, marker discovery aims to identify loci that harbor the greatest possible variation, with an eye toward capturing both within‐ and among‐population variation. In our study, we used pairwise *F*_ST_ to identify markers that *minimize* variation within species while maximizing differences *among* species, with the expectation that at least some outlier loci would exhibit strong pairwise differentiation for all species pairs. We had a relatively small sampling of numerous species to begin with, so we expected that our initial screening would discover false positives. We did find that by screening more stringently, we could correct for some false positives: Our stringent screening approach yielded 83 SNPs, whereas the relaxed approach exhibited a higher rate of false positives.

We were not certain at the outset of the study that we would find sufficient numbers of fixed or nearly fixed SNPs to develop a barcode in oaks, which are renowned for hybridization and lineage sorting (Hipp, 2015). In our study, we found 40 apparently fixed SNPs unique to nine species (based on our admittedly small sampling within species) and 147 nearly fixed SNPs, as well as two fixed SNPs at the clade level that were useful for distinguishing southern species from northern species in our sample (the clade comprising *Quercus stellata* and its relatives; Table 2). We found very few fixed or nearly fixed SNPs in *Q. stellata* and *Q. macrocarpa,* probably for two reasons: in *Q. macrocarpa,* we started with a larger candidate pool in the RAD‐seq dataset (*N *=* *9 individuals), which may have rendered our F_ST_ threshold for SNP discovery too stringent; and both species are wide in geographic range and known to hybridize with numerous other white oak species (Burger, 1975; Hardin, 1975; Rushton, 1993; Stoynoff & Hess, 2002; Whittemore & Schaal, 1991). Furthermore, during SNP screening we observed SNPs that we initially judged to be fixed or nearly fixed SNPs in *Q. stellata* that also turned out to be present in individuals of *Q. alba, Q. montana,* and *Q. michauxii*. Despite this fact, our barcode set suffices to delimit even these species, although it may reduce our power to distinguish hybrids from relatively pure individuals in, for example, *Q. stellata*. Our ability to distinguish species is an important result, given that 10–20 loci were needed to reliably distinguish just two white oak species in a previous barcoding study using the same technology (Guichoux et al., 2011). With additional sampling of individuals, we may find more false‐positive loci. However, given that the toolkit presented here is effective at identifying samples beyond the initial RAD‐seq sequencing run, and apparently effective in distinguishing hybrids from genetically pure individuals, we expect it to be robust to additional sampling.

### 4.4 Hybridization in a mixed‐species horticultural collection

The cultivated white oaks from which we sampled acorns exhibited low rates of hybridization for most individuals. This finding is at some odds with the traditional view, still quite common, that sympatric white oaks are utterly promiscuous (Burger, 1975; Hardin, 1975; Van Valen, 1976) or at least genetically cryptic (Craft & Ashley, 2006) and the expectation of high rates of hybridization in genotyped acorns (Moran et al., 2012). The mother trees selected were primarily of species not native to The Morton Arboretum and surrounding area: *Q. michauxii*,* Q. montana*,* Q. stellata*,* Q. muehlenbergii,* and *Q. prinoides* (the latter two species are very distinct ecologically and morphologically, but they are not distinguishable genetically in this study nor in a broader sample we have been working on for other projects). While this portion of the study was designed to demonstrate the barcode's potential for hybridization studies in mixed‐species collections of cultivated oaks, and our sample size was small, the preliminary findings are suggestive that both landscape position and possible reproductive barriers at the individual level play a role in hybridization patterns. For example, the mother tree that demonstrated the largest amount of hybridization was a *Q. michauxii* growing in close proximity to another mature *Q. michauxii*. In such a case, we might expect very limited hybridization (cf. Lagache et al., 2013). However, this *Q. michauxii* individual is also relatively close to the margin of a natural white oak forest, and it may be that in such a case, intraspecific pollination was swamped by interspecific pollination from the adjacent forest.

The *Q. stellata* offspring we genotyped yielded an interesting result. The mother tree, unexpectedly, appears to have been an F_1_ or early‐generation backcross with *Q. alba*, but the offspring all appear to be nearly pure *Q. alba* based on the STRUCTURE analysis. Our results suggest that the mother tree of these offspring is a hybrid that mates preferentially with white oak, although this result bears additional study. This result is not outlandish, as the individuals sampled are all adjacent to a large forest with large numbers of *Q. alba*, and *Q. stellata* is known to hybridize with *Q. alba* (Whittemore & Schaal, 1991). However, our finding that all acorns cluster predominantly with *Q. alba* may suggest that the number of *Q. stellata* SNPs we developed is simply too low to distinguish backcrosses from pure individuals. Of the 66 loci analyzed using STRUCTURE, only three were designed to identify *Q. alba*, and one of these was a nearly fixed SNP; and 12 were designed to identify *Q. stellata*, but all 12 were nearly fixed SNPs. Additional genotyping, ideally of controlled crosses, will be helpful what power we have to distinguish between pure individuals, F_1_ hybrids, and backcrosses between at least these two species.

By contrast, the *Q. prinoides* mother included in this study is a low‐growing shrub planted in a cluster with two others of its species and substantially further from the forest margin than the *Q. michauxii* mother tree discussed above. None of its acorns appear to be hybrids (Figure 3). It may well be that landscape position plays an important role in hybridization patterns in this garden. However, generalizing our findings will take a more thorough study of numerous mother trees over a number of years, evaluating landscape position relative to other species. The barcoding presented here should facilitate such studies.

## 5 CONCLUDING REMARKS

Living collections may be the last harbor for many endangered trees and important repositories of genetic diversity, but only if planting strategies and management are optimized to control gene flow and genetic diversity (Cavender‐Bares et al., 2015). DNA barcoding can be an effective tool to quickly assess whether newly introduced endangered species are susceptible to hybridization with native populations, but DNA barcoding is often problematic in trees due to incomplete lineage sorting and hybridization (Arca et al., 2012; Percy et al., 2014; Pham et al., 2017; Simeone et al., 2013). In this study, we solve the problem of DNA barcoding for a regional sample of interbreeding white oaks, producing a novel genotyping tool that works to identify species and their hybrids. The developed barcoding toolkit should be useful in predicting which species are more likely to hybridize, monitoring hybridization in response to climate change, and ultimately predicting how species introductions and planting strategies will impact hybridization in artificial plantings. Such attention to genetic conservation will help to guide and manage future plantings of oaks in common gardens, living collections, and elsewhere.

## CONFLICT OF INTEREST

None declared.

## AUTHORS’ CONTRIBUTIONS

ALH and EF conceived and designed the study and formulated analyses. ALH and MH generated and analyzed the RAD‐seq dataset. ALH developed analytical tools to identify SNPs in the clustered RAD‐seq loci. EF extracted DNA from acorns. EG performed primer development. AD performed the MassARRAY experiments. EF performed bioinformatics queries for in silico SNP discovery, analyzed the data, and performed barcode evaluation. ML made the fine‐scale map of the study site at The Morton Arboretum. EF and ALH wrote the manuscript with the help of all authors. All authors have checked and approved the final version of the manuscript.

## DATA ACCESSIBILITY

FASTQ (RAD‐seq) files are available under NCBI BioProject PRJNA376740 (https://www.ncbi.nlm.nih.gov/bioproject/376740). Scripts and auxiliary data utilized in this article are available from the Dryad Digital Repository: https://doi.org/10.5061/dryad.dg5hv06.

## References

[ece34122-bib-0001] Alberto, F. J. , Derory, J. , Boury, C. , Frigerio, J. M. , Zimmermann, N. E. , & Kremer, A. (2013). Imprints of natural selection along environmental gradients in phenology‐related genes of *Quercus petraea* . Genetics, 195, 495–512.https://doi.org/10.1534/genetics.113.153783 23934884 10.1534/genetics.113.153783 PMC3781976

[ece34122-bib-0002] Altschul, S. F. , Gish, W. , Miller, W. , Myers, E. W. , & Lipman, D. J. (1990). Basic local alignment search tool. Journal of Molecular Biology, 215, 403–410. https://doi.org/10.1016/S0022-2836(05)80360-2 2231712 10.1016/S0022-2836(05)80360-2

[ece34122-bib-0003] Appleby, N. , Edwards, D. , & Batley, J. (2009). New technologies for ultra‐high throughput genotyping in plants In Somers D. G. (Ed.), Plant genomics (pp. 19–39). New York, NY: Humana Press https://doi.org/10.1007/978-1-59745-427-8 10.1007/978-1-59745-427-8_2 19347650

[ece34122-bib-0004] Arca, M. , Hinsinger, D. D. , Cruaud, C. , Tillier, A. , Bousquet, J. , & Frascaria‐Lacoste, N. (2012). Deciduous trees and the application of universal DNA barcodes: A case study on the circumpolar fraxinus. PLoS ONE, 7, e34089 https://doi.org/10.1371/journal.pone.0034089 22479532 10.1371/journal.pone.0034089 PMC3313964

[ece34122-bib-0005] Baird, N. A. , Etter, P. D. , Atwood, T. S. , Currey, M. C. , Shiver, A. L. , Lewis, Z. A. , … Johnson, E. A. (2008). Rapid SNP discovery and genetic mapping using sequenced RAD markers. PLoS ONE, 3, e3376 https://doi.org/10.1371/journal.pone.0003376 18852878 10.1371/journal.pone.0003376 PMC2557064

[ece34122-bib-0006] Barchi, L. , Lanteri, S. , Portis, E. , Acquadro, A. , Valè, G. , Toppino, L. , & Rotino, G. L. (2011). Identification of SNP and SSR markers in eggplant using RAD tag sequencing. BMC Genomics, 12, 304. 21663628 10.1186/1471-2164-12-304 PMC3128069

[ece34122-bib-0007] Bradić, M. , Costa, J. , & Chelo, I. M. (2012). Genotyping with Sequenom In Orgogozo V., & Rockman M. (Eds.), Molecular methods for evolutionary genetics, vol. 772. Methods in Molecular Biology (Methods and Protocols) (pp. 192–210). New York, NY: Humana Press. 10.1007/978-1-61779-228-1_11 22065439

[ece34122-bib-0008] Burgarella, C. , Lorenzo, Z. , Jabbour‐Zahab, R. , Lumaret, R. , Guichoux, E. , Petit, R. J. , … Gil, L. (2009). Detection of hybrids in nature: Application to oaks (*Quercus suber* and *Q. ilex*). Heredity, 102, 442–452. https://doi.org/10.1038/hdy.2009.8 19240752 10.1038/hdy.2009.8

[ece34122-bib-0009] Burger, W. C. (1975). The species concept in *Quercus* . Taxon, 24, 45–50. https://doi.org/10.2307/1218998

[ece34122-bib-0010] Catchen, J. M. , Amores, A. , Hohenlohe, P. , Cresko, W. , & Postlethwait, J. H. (2011). Stacks: Building and genotyping loci de novo from short‐read sequences. G3: Genes, Genomes, Genetics, 1, 171–182. https://doi.org/10.1534/g3.111.000240 22384329 10.1534/g3.111.000240 PMC3276136

[ece34122-bib-0011] Catchen, J. , Hohenlohe, P. A. , Bassham, S. , Amores, A. , & Cresko, W. A. (2013). Stacks: An analysis tool set for population genomics. Molecular Ecology, 22, 3124–3140. https://doi.org/10.1111/mec.12354 23701397 10.1111/mec.12354 PMC3936987

[ece34122-bib-0012] Cavender‐Bares, J. (2016). Diversity, distribution and ecosystem services of the North American Oaks. International Oaks, 27, 37–48.

[ece34122-bib-0013] Cavender‐Bares, J. , González‐Rodríguez, . , Eaton, D. A. , Hipp, A. A. , Beulke, A. , & Manos, P. S. (2015). Phylogeny and biogeography of the American live oaks (Quercus subsection Virentes): A genomic and population genetics approach. Molecular Ecology, 24, 3668–3687. https://doi.org/10.1111/mec.13269 26095958 10.1111/mec.13269

[ece34122-bib-0014] Chagné, D. , Lalanne, C. , Madur, D. , Kumar, S. , & Frigério, J. (2002). A high density genetic map of maritime pine based on AFLPs. Annales of Forest Science, 59, 627–636. https://doi.org/10.1051/forest:2002048

[ece34122-bib-0015] Chancerel, E. , Lepoittevin, C. , Le Provost, G. , Lin, Y. C. , Jaramillo‐ Correa, J. P. , Eckert, A. J. , … Chaumeil, P. (2011). Development and implementation of a highly‐multiplexed SNP array for genetic mapping in maritime pine and comparative mapping with loblolly pine. BMC Genomics, 12, 368 https://doi.org/10.1186/1471-2164-12-368 21767361 10.1186/1471-2164-12-368 PMC3146957

[ece34122-bib-0016] Chen, J. , Zeng, Y.‐F. , Liao, W.‐J. , Yan, P.‐C. , & Zhang, J.‐G. (2017). A novel set of single‐copy nuclear gene markers in white oak and implications for species delimitation. Tree Genetics & Genomes, 13, 50 https://doi.org/10.1007/s11295-017-1130-3

[ece34122-bib-0017] Chybicki, I. J. , & Burczyk, J. (2010). Realized gene flow within mixed stands of *Quercus robur* L. and *Q. petraea* (Matt.) L. revealed at the stage of naturally established seedling. Molecular Ecology, 19, 2137–2151. https://doi.org/10.1111/j.1365-294X.2010.04632.x 20550635 10.1111/j.1365-294X.2010.04632.x

[ece34122-bib-0018] Craft, K. J. , & Ashley, M. V. (2006). Population differentiation among three species of white oak in northeastern Illinois. Canadian Journal of Forest Research, 26, 206–215. https://doi.org/10.1139/x05-234

[ece34122-bib-0019] Curtu, A. L. , Gailing, O. , & Finkeldey, R. (2007). Evidence for hybridization and introgression within a species‐rich oak (*Quercus* spp.) community. BMC Evolutionary Biology, 7, 218–232. https://doi.org/10.1186/1471-2148-7-218 17996115 10.1186/1471-2148-7-218 PMC2244923

[ece34122-bib-0020] Davey, J. L. , & Blaxter, M. W. (2010). RADseq: Next‐generation population genetics. Briefings in Functional Genomics, 9, 416–423. https://doi.org/10.1093/bfgp/elq031 21266344 10.1093/bfgp/elq031 PMC3080771

[ece34122-bib-0021] Denk, T. , & Grimm, G. W. (2010). The oaks of western Eurasia: Traditional classifications and evidence from two nuclear markers. Taxon, 59, 351–366.

[ece34122-bib-0022] Dereeper, A. , Guignon, V. , Blanc, G. , Audic, S. , Buffet, S. , Chevenet, F. , … Claverie, J. M. (2008). Phylogeny.fr: Robust phylogenetic analysis for the non‐specialist. Nucleic Acids Research, 36, W465–W469. https://doi.org/10.1093/nar/gkn180 18424797 10.1093/nar/gkn180 PMC2447785

[ece34122-bib-0023] Dodd, R. S. , & Afzal‐Rafii, Z. (2004). Selection and dispersal in a multispecies oak hybrid zone. Evolution, 58, 261–269. https://doi.org/10.1111/j.0014-3820.2004.tb01643.x 15068344

[ece34122-bib-0024] Dumolin‐Lapègue, S. , Démesure, B. , Fineschi, S. , Le Corre, V. , & Petit, R. J. (1997). Phylogeographic structure of white oaks throughout the European continent. Genetics, 146, 1475–1487. 9258689 10.1093/genetics/146.4.1475 PMC1208090

[ece34122-bib-0025] Dumolin‐Lapègue, S. , Kremer, A. , & Petit, R. J. (1999). Are chloroplast and mitochondrial DNA variation species independent in oaks? Evolution, 53, 1406–1413. https://doi.org/10.1111/j.1558-5646.1999.tb05405.x 28565571 10.1111/j.1558-5646.1999.tb05405.x

[ece34122-bib-0026] Durand, J. , Bodénès, C. , Chancerel, E. , Frigerio, J. M. , Vendramin, G. , Sebastiani, F. , … Mattioni, C. (2010). A fast and cost‐effective approach to develop and map EST‐SSR markers: Oak as a case study. BMC Genomics, 11, 570 https://doi.org/10.1186/1471-2164-11-570 20950475 10.1186/1471-2164-11-570 PMC3091719

[ece34122-bib-0027] Eaton, D. A. R. (2014). PyRAD: Assembly of de novo RADseq loci for phylogenetic analyses. Bioinformatics, 30, 1844–1849. https://doi.org/10.1093/bioinformatics/btu121 24603985 10.1093/bioinformatics/btu121

[ece34122-bib-0028] Eaton, D. A. R. , Hipp, A. L. , González‐Rodríguez, A. , & Cavender‐Bares, J. (2015). Historical introgression among the American live oaks and the comparative nature of tests for introgression. Evolution, 69, 2587–2601. https://doi.org/10.1111/evo.12758 26299374 10.1111/evo.12758

[ece34122-bib-0029] Edgar, R. C. (2010). Search and clustering orders of magnitude faster than BLAST. Bioinformatics, 26, 2460–2461. https://doi.org/10.1093/bioinformatics/btq461 20709691 10.1093/bioinformatics/btq461

[ece34122-bib-0030] Efrain Tovar‐Sanchez, K. O. (2004). Natural hybridization and hybrid zones between *Quercus Crassifolia* and *Quercus Crassipes* (Fagaceae) in Mexico: Morphological and molecular evidence. American Journal of Botany, 91, 1352–1363. https://doi.org/10.3732/ajb.91.9.1352 21652368 10.3732/ajb.91.9.1352

[ece34122-bib-0031] Evanno, G. , Regnaut, S. , & Goudet, J. (2005). Detecting the number of clusters of individuals using the software structure: A simulation study. Molecular Ecology, 14, 2611–2620. https://doi.org/10.1111/j.1365-294X.2005.02553.x 15969739 10.1111/j.1365-294X.2005.02553.x

[ece34122-bib-0032] Fitz‐Gibbon, S. , Hipp, A. L. , Pham, K. K. , Manos, P. S. , & Sork, V. S. (2017). Phylogenomic inferences from reference‐mapped and de novo assembled short‐ read sequence data using RADseq sequencing of California white oaks (*Quercus* subgenus *Quercus*). Genome, 60, 743–755. https://doi.org/10.1139/gen-2016-0202 28355490 10.1139/gen-2016-0202

[ece34122-bib-0033] Gerber, S. , Chadoeuf, J. , Gugerli, F. , Lascoux, M. , Buiteveld, J. , Cottrell, J. , … Goicoechea, P. G. (2014). High rates of gene flow by pollen and seed in oak populations across Europe. PLoS ONE, 9, e85130 https://doi.org/10.1371/journal.pone.0085130 24454802 10.1371/journal.pone.0085130 PMC3890301

[ece34122-bib-0034] Goudet, J. (2013) Estimation and tests of hierarchical F‐statistics. R package version 0.04‐10. http://CRAN.R-project.org/package=hierfstat.

[ece34122-bib-0035] Guichoux, E. , Lagache, L. , Wagner, S. , Léger, P. , & Petit, R. J. (2011). Two highly validated multiplexes (12‐plex and 8‐plex) for species delimitation and parentage analysis in oaks (*Quercus* spp.). Molecular Ecology Resources, 11, 578–585. https://doi.org/10.1111/j.1755-0998.2011.02983.x 21481218 10.1111/j.1755-0998.2011.02983.x

[ece34122-bib-0036] Guindon, S. , Dufayard, J.‐F. , Lefort, V. , Anisimova, M. , Hordijk, W. , & Gascuel, O. (2010). New algorithms and methods to estimate maximum‐likelihood phylogenies: Assessing the performance of PhyML 3.0. Systematic Biology, 59, 307–321. https://doi.org/10.1093/sysbio/syq010 20525638 10.1093/sysbio/syq010

[ece34122-bib-0037] Hardin, J. W. (1975). Hybridization and introgression in *Quercus alba* . Journal of the Arnold Arboretum, 56, 336–363.

[ece34122-bib-0038] Hasselman, D. J. , Argo, E. E. , McBride, M. C. , Bentzen, P. , Schultz, T. F. , Perez‐ Umphrey, A. A. , & Palkovacs, E. P. (2014). Human disturbance causes the formation of a hybrid swarm between two naturally sympatric fish species. Molecular Ecology, 23, 1137–1152. https://doi.org/10.1111/mec.12674 24450302 10.1111/mec.12674

[ece34122-bib-0039] Hegarty, M. J. , Barker, G. L. , Brennan, A. C. , Edwards, K. J. , Abbott, R. J. , & Hiscock, S. J. (2008). Changes to gene expression associated with hybrid speciation in plants: Further insights from transcriptomic studies in *Senecio* . Philosophical Transactions of the Royal Society of London Series B, Biological Sciences, 363, 3055–3069. https://doi.org/10.1098/rstb.2008.0080 18579474 10.1098/rstb.2008.0080 PMC2607317

[ece34122-bib-0040] Heslot, N. , Rutkoski, J. , Poland, J. , Jannink, J. L. , & Sorrells, M. E. (2013). Impact of marker ascertainment bias on genomic selection accuracy and estimates of genetic diversity. PLoS ONE, 8, e74612 https://doi.org/10.1371/journal.pone.0074612 24040295 10.1371/journal.pone.0074612 PMC3764096

[ece34122-bib-0041] Hipp, A. L. (2015). Should hybridization make us skeptical of the oak phylogeny ? International Oaks, 26, 9–18.

[ece34122-bib-0042] Hipp, A. L. , Eaton, D. A. , Cavender‐Bares, J. , Fitzek, E. , Nipper, R. , & Manos, P. S. (2014). A framework phylogeny of the American oak clade based on sequenced RAD data. PLoS ONE, 9, e93975 https://doi.org/10.1371/journal.pone.0093975 24705617 10.1371/journal.pone.0093975 PMC3976371

[ece34122-bib-0043] Hipp, A. L. , Manos, P. S. , Gonzalez‐Rodriguez, A. , Hahn, M. , Kaproth, M. , McVay, J. D. , … Cavender‐Bares, J. (2018). Sympatric parallel diversification of major oak clades in the Americas and the origins of Mexican oak diversity. New Phytologist, 217, 439–452. https://doi.org/10.1111/nph.14773 28921530 10.1111/nph.14773

[ece34122-bib-0044] Hipp, A. L. , & Weber, J. A. (2008). Taxonomy of Hill's Oak (*Quercus ellipsoidalis*: Fagaceae): Evidence from AFLP Data. Systematic Botany, 33, 148–158. https://doi.org/10.1600/036364408783887320

[ece34122-bib-0045] Hoban, S. M. , Mccleary, T. S. , Schlarbaum, S. E. , Anagnostakis, S. L. , & Romero‐Severson, J. (2012). Human‐impacted landscapes facilitate hybridization between a native and an introduced tree. Evolutionary Applications, 5, 720–731. https://doi.org/10.1111/j.1752-4571.2012.00250.x 23144658 10.1111/j.1752-4571.2012.00250.x PMC3492897

[ece34122-bib-0046] Hollingsworth, P. M. , Graham, S. W. , & Little, D. P. (2011). Choosing and using a plant DNA barcode. PLoS ONE, 6, e19254 https://doi.org/10.1371/journal.pone.0019254 21637336 10.1371/journal.pone.0019254 PMC3102656

[ece34122-bib-0047] Hubert, F. , Grimm, G. W. , Jousselin, E. , Berry, V. , Franc, A. , & Kremer, A. (2014). Multiple nuclear genes stabilize the phylogenetic backbone of the genus *Quercus* . Systematics and Biodiversity, 12, 405–423. https://doi.org/10.1080/14772000.2014.941037

[ece34122-bib-0048] Hulme, P. E. (2015). Resolving whether botanic gardens are on the road to conservation or a pathway for plant invasions. Conservation Biology, 29, 816–824. https://doi.org/10.1111/cobi.12426 25439324 10.1111/cobi.12426

[ece34122-bib-0049] Jiggins, C. D. , Salazar, C. , Linares, M. , & Mavarez, J. (2008). Hybrid trait speciation and *Heliconius* butterflies. Philosophical Transactions of the Royal Society of London, Series B, Biological sciences, 363, 3047–3054. https://doi.org/10.1098/rstb.2008.0065 18579480 10.1098/rstb.2008.0065 PMC2607310

[ece34122-bib-0050] Kearse, M. , Moir, R. , Wilson, A. , Stones‐Havas, S. , Cheung, M. , Sturrock, S. , … Thierer, T. (2012). Geneious Basic: An integrated and extendable desktop software platform for the organization and analysis of sequence data. Bioinformatics, 28, 1647–1649. https://doi.org/10.1093/bioinformatics/bts199 22543367 10.1093/bioinformatics/bts199 PMC3371832

[ece34122-bib-0051] Kress, W. J. , Erickson, D. L. , Jones, F. A. , Swenson, N. G. , Perez, R. , Sanjur, O. , & Bermingham, E. (2009). Plant DNA barcodes and a community phylogeny of a tropical forest dynamics plot in Panama. Proceedings of the National Academy of Sciences, 106(44), 18621–18626. https://doi.org/10.1073/pnas.0909820106 10.1073/pnas.0909820106 PMC2763884 19841276

[ece34122-bib-0052] Kress, W. J. , García‐Robledo, C. , Uriarte, M. , & Erickson, D. L. (2015). DNA barcodes for ecology, evolution, and conservation. Trends in Ecology & Evolution, 30(1), 25–35. https://doi.org/10.1016/j.tree.2014.10.008 25468359 10.1016/j.tree.2014.10.008

[ece34122-bib-0053] Lagache, L. , Klein, E. K. , Guichoux, E. , & Petit, R. J. (2013). Fine‐scale environmental control of hybridization in oaks. Molecular Ecology, 22, 423–436. https://doi.org/10.1111/mec.12121 23173566 10.1111/mec.12121

[ece34122-bib-0054] Larkin, D. J. (2012). Lengths and correlates of lag phases in upper‐Midwest plant invasions. Biological Invasions, 14, 827–838. https://doi.org/10.1007/s10530-011-0119-3

[ece34122-bib-0055] Letunic, I. , & Bork, P. (2016). Interactive tree of life (iTOL) v3: An online tool for the display and annotation of phylogenetic and other trees. Nucleic Acids Research, 44, W242–W245. https://doi.org/10.1093/nar/gkw290 27095192 10.1093/nar/gkw290 PMC4987883

[ece34122-bib-0056] Lexer, C. , Kremer, A. , & Petit, R. J. (2006). Shared alleles in sympatric oaks: Recurrent gene flow is a more parsimonious explanation than ancestral polymorphism. Molecular Ecology, 15, 2007–2012. https://doi.org/10.1111/j.1365-294X.2006.02896.x 16689915 10.1111/j.1365-294X.2006.02896.x

[ece34122-bib-0057] Lijavetzky, D. , Cabezas, J. A. , Ibáñez, A. , Rodríguez, V. , & Martínez‐Zapater, J. M. (2007). High throughput SNP discovery and genotyping in grapevine (*Vitis vinifera* L.) by combining a re‐sequencing approach and SNPlex technology. BMC Genomics, 8, 424 https://doi.org/10.1186/1471-2164-8-424 18021442 10.1186/1471-2164-8-424 PMC2212664

[ece34122-bib-0058] Lynch, M. (2008). Estimation of nucleotide diversity, disequilibrium coefficients, and mutation rates from high‐coverage genome‐sequencing projects. Molecular Biology and Evolution, 25, 2409–2419. https://doi.org/10.1093/molbev/msn185 18725384 10.1093/molbev/msn185 PMC2767098

[ece34122-bib-0059] Mallet, J. (2007). Hybrid speciation. Nature, 446, 279–283. https://doi.org/10.1038/nature05706 17361174 10.1038/nature05706

[ece34122-bib-0060] McVay, J. , Hauser, D. , Hipp, A. L. , & Manos, P. S. (2017). Phylogenomics reveals a complex evolutionary history of lobed‐leaf white oaks in Western North America. Genome, 60, 733–742. https://doi.org/10.1139/gen-2016-0206 28727934 10.1139/gen-2016-0206

[ece34122-bib-0061] McVay, J. D. , Hipp, A. L. , & Manos, P. S. (2017). A genetic legacy of introgression confounds phylogeny and biogeography in oaks. Proceedings of the Royal Society B: Biological Sciences, 284, 20170300 https://doi.org/10.1098/rspb.2017.0300 28515204 10.1098/rspb.2017.0300 PMC5443950

[ece34122-bib-0062] Meng, C. , & Kubatko, L. S. (2009). Detecting hybrid speciation in the presence of incomplete lineage sorting using gene tree incongruence: A model. Theoretical Population Biology, 75, 35–45. https://doi.org/10.1016/j.tpb.2008.10.004 19038278 10.1016/j.tpb.2008.10.004

[ece34122-bib-0063] Moran, E. V. , Willis, J. , & Clark, J. S. (2012). Genetic evidence for hybridization in red oaks (*Quercus* sect. *Lobatae*, Fagaceae). American Journal of Botany, 99, 92–100. https://doi.org/10.3732/ajb.1100023 22174334 10.3732/ajb.1100023

[ece34122-bib-0064] Morgan, M. , Anders, S. , Lawrence, M. , Aboyoun, P. , Pages, H. , & Gentleman, R. (2009). ShortRead: A bioconductor package for input, quality assessment and exploration of high‐throughput sequence data. Bioinformatics, 25, 2607–2608. https://doi.org/10.1093/bioinformatics/btp450 19654119 10.1093/bioinformatics/btp450 PMC2752612

[ece34122-bib-0065] Novaes, E. , Drost, D. R. , Farmerie, W. G. , Pappas, G. J. , Grattapaglia, D. , Sederoff, R. R. , & Kirst, M. (2008). High‐throughput gene and SNP discovery in *Eucalyptus grandis*, an uncharacterized genome. BMC Genomics, 9, 312 https://doi.org/10.1186/1471-2164-9-312 18590545 10.1186/1471-2164-9-312 PMC2483731

[ece34122-bib-0066] Oh, S. H. , & Manos, P. S. (2008). Molecular phylogenetics and cupule evolution in Fagaceae as inferred from nuclear CRABS CLAW sequences. Taxon, 57, 434–451.

[ece34122-bib-0067] Owusu, S. A. , Sullivan, A. R. , Weber, J. A. , Hipp, A. L. , & Gailing, O. (2015). Taxonomic relationships and gene flow in four North American *Quercus* Species (*Quercus* section *Lobatae*). Systematic Botany, 40, 510–521. https://doi.org/10.1600/036364415X688754

[ece34122-bib-0068] Pegadaraju, V. , Nipper, R. , Hulke, B. , Qi, L. , & Schultz, Q. (2013). De novo sequencing of sunflower genome for SNP discovery using RAD (Restriction site Associated DNA) approach. BMC Genomics, 14, 556 https://doi.org/10.1186/1471-2164-14-556 23947483 10.1186/1471-2164-14-556 PMC3765701

[ece34122-bib-0069] Peñaloza‐Ramírez, J. M. , González‐Rodríguez, A. , Mendoza‐Cuenca, L. , Caron, H. , Kremer, A. , & Oyama, K. (2010). Interspecific gene flow in a multispecies oak hybrid zone in the Sierra Tarahumara of Mexico. Annals of Botany, 105, 389–399. https://doi.org/10.1093/aob/mcp301 20056653 10.1093/aob/mcp301 PMC2826251

[ece34122-bib-0070] Percy, D. M. , Argus, G. W. , Cronk, Q. C. , Fazekas, A. J. , Kesanakurti, P. R. , Burgess, K. S. , … Graham, S. W. (2014). Understanding the spectacular failure of DNA barcoding in willows (*Salix*): Does this result from a trans‐specific selective sweep? Molecular Ecology, 23, 4737–4756. https://doi.org/10.1111/mec.12837 24944007 10.1111/mec.12837

[ece34122-bib-0071] Petit, R. J. (1993). Gene diversity in natural populations of oak species. Annals of Forest Science, 50, 186s–202s.

[ece34122-bib-0072] Petit, R. J. , Bodénès, C. , Ducousso, A. , Roussel, G. , & Kremer, A. (2004). Hybridization as a mechanism of invasion in oaks. New Phytologist, 161, 151–164.

[ece34122-bib-0073] Pham, K. K. , Hipp, A. L. , Manos, P. S. , & Cronn, R. C. (2017). A time and a place for everything: Phylogenetic history and geography as joint predictors of oak plastome phylogeny. Genome, 60, 720–732. https://doi.org/10.1139/gen-2016-0191 28445658 10.1139/gen-2016-0191

[ece34122-bib-0074] Piredda, R. , Simeone, M. C. , Attimonelli, M. , Bellarosa, R. , & Schirone, B. (2011). Prospects of barcoding the Italian wild dendroflora: Oaks reveal severe limitations to tracking species identity. Molecular Ecology Resources, 11, 72–83. https://doi.org/10.1111/j.1755-0998.2010.02900.x 21429102 10.1111/j.1755-0998.2010.02900.x

[ece34122-bib-0075] Prasad, A. M. , Gardiner, J. D. , Iverson, L. R. , Matthews, S. N. , & Peters, M. (2013). Exploring tree species colonization potentials using a spatially explicit simulation model: Implications for four oaks under climate change. Global Change Biology, 19, 2196–2208. https://doi.org/10.1111/gcb.12204 23526802 10.1111/gcb.12204

[ece34122-bib-0076] Pritchard, J. K. , Stephens, M. , & Donnelly, P. (2000). Inference of population structure using multilocus genotype data. Genetics, 155, 945–959. 10835412 10.1093/genetics/155.2.945 PMC1461096

[ece34122-bib-0077] Putman, A. I. , & Carbone, I. (2014). Challenges in analysis and interpretation of microsatellite data for population genetic studies. Ecology and Evolution, 4, 4399–4428. 25540699 10.1002/ece3.1305 PMC4267876

[ece34122-bib-0078] R Core Team (2016). R: A language and environment for statistical computing. Vienna, Austria: R Foundation for Statistical Computing URL: https://www.R-project.org/.

[ece34122-bib-0079] Reichard, S. H. , & White, P. (2001). Horticulture as a pathway of invasive plant introductions in the United States. BioScience, 51, 103–113. https://doi.org/10.1641/0006-3568(2001)051[0103:HAAPOI]2.0.CO;2

[ece34122-bib-0080] Rosenberg, N. A. (2004). DISTRUCT: A program for the graphical display of population structure. Molecular Ecology Notes, 4, 137–138.

[ece34122-bib-0081] Rushton, B. S. (1993). Natural hybridization within the genus *Quercus* L. Annals of Forest Science, 50, 73–90. https://doi.org/10.1051/forest:19930707

[ece34122-bib-0082] Scaglione, D. , Acquadro, A. , Portis, E. , Tirone, M. , Knapp, S. J. , & Lanteri, S. (2012). RAD tag sequencing as a source of SNP markers in *Cynara cardunculus* L. BMC Genomics, 13, 3 https://doi.org/10.1186/1471-2164-13-3 22214349 10.1186/1471-2164-13-3 PMC3269995

[ece34122-bib-0083] Schumer, M. , Rosenthal, G. G. , & Andolfatto, P. (2014). How common is homoploid hybrid speciation? Evolution, 68, 1553–1560. https://doi.org/10.1111/evo.12399 24620775 10.1111/evo.12399

[ece34122-bib-0084] Seehausen, O. , Takimoto, G. , Roy, D. , & Jokela, J. (2008). Speciation reversal and biodiversity dynamics with hybridization in changing environments. Molecular Ecology, 17, 30–44. https://doi.org/10.1111/j.1365-294X.2007.03529.x 18034800 10.1111/j.1365-294X.2007.03529.x

[ece34122-bib-0085] Simeone, M. C. , Piredda, R. , Papini, A. , Vessella, F. , & Schirone, B. (2013). Application of plastid and nuclear markers to DNA barcoding of Euro‐Mediterranean oaks (*Quercus*, Fagaceae): Problems, prospects and phylogenetic implications. Botanical Journal of the Linnean Society, 172, 478–499. https://doi.org/10.1111/boj.12059

[ece34122-bib-0086] Song, Y. , Deng, M. , Hipp, A. L. , & Li, Q. (2015). Leaf morphological evidence of natural hybridization between two oak species (*Quercus austrocochinchinensis* and *Q. kerrii*) and its implications for conservation management. European Journal of Forest Research, 134, 139–151. https://doi.org/10.1007/s10342-014-0839-x

[ece34122-bib-0087] Stamatakis, A. , Hoover, P. , & Rougemont, J. (2008). A rapid bootstrap algorithm for the RAxML Web servers. Systematic Biology, 57, 758–771. https://doi.org/10.1080/10635150802429642 18853362 10.1080/10635150802429642

[ece34122-bib-0088] Stoynoff, N. A. , & Hess, W. J. (2002). The distribution of the genus *Quercus* in Illinois: An update. Transactions of the Illinois State Academy of Science, 95, 261–284.

[ece34122-bib-0089] Sullivan, A. R. , Owusu, S. A. , Weber, J. A. , Hipp, A. L. , & Gailing, O. (2016). Hybridization and divergence in multi‐species oak (*Quercus*) communities. Botanical Journal of the Linnean Society, 181, 99–114. https://doi.org/10.1111/boj.12393

[ece34122-bib-0090] The Global *Carex* Group , Jiménez‐Mejías, P. , Hahn, M. , Lueders, K. , Starr, J. R. , Brown, B. H. , Chouinard, B. N. , … Roalson, E. H. (2016). Megaphylogenetic specimen‐level approaches to the *Carex* (Cyperaceae) phylogeny using ITS, ETS, and matK sequences: Implications for classification. Systematic Botany, 41, 500–518.

[ece34122-bib-0091] Twyford, A. D. , & Ennos, R. A. (2012). Next‐generation hybridization and introgression. Heredity, 108, 179–189. https://doi.org/10.1038/hdy.2011.68 21897439 10.1038/hdy.2011.68 PMC3282392

[ece34122-bib-0092] Unger, G. M. , Vendramin, G. G. , & Robledo‐Arnuncio, J. J. (2014). Estimating exotic gene flow into native pine stands: Zygotic vs. gametic components. Molecular Ecology, 23, 5435–5447. https://doi.org/10.1111/mec.12946 25277767 10.1111/mec.12946

[ece34122-bib-0093] Van Droogenbroeck, B. , Kyndt, T. , Maertens, I. , Romeijn‐Peeters, E. , Scheldeman, X. , Romero‐ Motochi, J. P. , … Gheysen, G. (2004). Phylogenetic analysis of the highland papayas (*Vasconcellea*) and allied genera (Caricaceae) using PCR‐RFLP. Theoretical and Applied Genetics, 108, 1473–1486. https://doi.org/10.1007/s00122-003-1575-7 14752605 10.1007/s00122-003-1575-7

[ece34122-bib-0094] Van Valen, L. (1976). Ecological species, multispecies, and oaks. Taxon, 25, 233–239. https://doi.org/10.2307/1219444

[ece34122-bib-0095] Wei, L. , Li, Y. F. , Zhang, H. , & Liao, W. J. (2015). Variation in morphological traits in a recent hybrid zone between closely related *Quercus liaotungensis* and *Q. mongolica* (Fagaceae). Journal of Plant Ecology, 8, 224–229. https://doi.org/10.1093/jpe/rtv023

[ece34122-bib-0096] Whittemore, A. T. , & Schaal, B. A. (1991). interspecific gene flow in sympatric oaks. Proceedings of the National Academy of Sciences of the United States of America, 88, 2540–2544. https://doi.org/10.1073/pnas.88.6.2540 11607170 10.1073/pnas.88.6.2540 PMC51268

[ece34122-bib-0097] Yan, J. , Yang, X. , Shah, T. , Sánchez‐Villeda, H. , Li, J. , Warburton, M. , … Xu, Y. (2010). High‐throughput SNP genotyping with the Goldengate assay in Maize. Molecular Breeding, 25, 441–451. https://doi.org/10.1007/s11032-009-9343-2

[ece34122-bib-0098] Yang, J. , Vázquez, L. , Chen, X. , Li, H. , Zhang, H. , Liu, Z. , & Zhao, G. (2017). Development of chloroplast and nuclear DNA markers for Chinese Oaks (*Quercus* Subgenus *Quercus*) and assessment of their utility as DNA barcodes. Frontiers in Plant Science, 8, 816 https://doi.org/10.3389/fpls.2017.00816 28579999 10.3389/fpls.2017.00816 PMC5437370

[ece34122-bib-0099] Zaya, D. N. , Leicht‐Young, S. A. , Pavlovic, N. B. , Feldheim, K. A. , & Ashley, M. V. (2015). Genetic characterization of hybridization between native and invasive bittersweet vines (*Celastrus* spp.). Biological Invasions, 17, 2975–2988. https://doi.org/10.1007/s10530-015-0926-z

