# Supplementary material for: A nuclear DNA barcode for eastern North American oaks and application to a study of hybridization in an Arboretum setting
Source: Ecol Evol. 2018 May 8;8(11):5837–51. doi: 10.1002/ece3.4122 (PMC6010771; doi:10.1002/ece3.4122)
Supplement: Supplementary file 1 [file ECE3-8-5837-s001.pdf]

Supporting Information

Supplementary files

Supplementary file 1. SNP evaluation. Accession and species identification of samples used to evaluate potential SNPs. Samples that were also used for SNP identification and served as learning samples during barcode evaluation are indicated with ‘x’. Samples not coinciding with SNP identification and learning samples are indicated with ‘- -‘.

Supplementary file 2. Custom script to select for species-specific SNP per locus sequence (PyRAD/RADami approach).

Supplementary file 3. Custom script to select for species-specific SNPs per locus sequence (STACKS approach).

Supplementary file 4. Barcode selection. Out of 291 screened SNPs at least 119 SNPs are considered for barcode genotyping tool kit. Maximum likelihood (Phylogeny.fr) was used to verify SNP selection.

Supplementary file 5. List of 70 SNP-multiplex. Selected SNPs for 2-plex with SNP_ID corresponding species and RAD-seq with implemented polymorphism. SNP_IDs in bold = 1st barcode multiplex. The remaining SNPs_IDs belong to 2nd multiplex of the barcode. Table is sorted alphabetically by species.
Clade 1 = *Q. sinuata*, *Q. oglethorpensis*, *Q. boyntonii*, *Q. stellata*, *Q. chapmanii*, *Q. austrina*. Asian white oaks = *Q. aliena*, *Q. fabri*. European white oaks = *Q. faginea*, *Q. petraea*, *Q. robur*.

Supplementary file 1. SNP evaluation.

Species	Accession id	SNP identification	Learning sample (barcode evaluation)
Quercus alba	OAK-MOR-130	--	x
Quercus alba	OAK-MOR-613	--	x
Quercus alba	OAK-MOR-204	x	x
Quercus alba	OAK-MOR-359	x	x
Quercus alba	OAK-MOR-76	x	x
Quercus alba	PM-19	x	x
Quercus aliena	OAK-MOR-698	--	x
Quercus austrina	OAK-MOR-530	--	--
Quercus austrina	OAK-MOR-511	x	--
Quercus bicolor	OAK-MOR-246	--	x
Quercus bicolor	OAK-MOR-289	x	x
Quercus bicolor	OAK-MOR-342	x	x
Quercus boyntonii	OAK-MOR-181	--	--
Quercus boyntonii	OAK-MOR-427	--	--
Quercus boyntonii	OAK-MOR-436	x	--
Quercus boyntonii	OAK-MOR-544	x	--
Quercus brandegeei	OAK-MOR-147	--	--
Quercus chapmanii	OAK-MOR-220	--	--
Quercus chapmanii	OAK-MOR-444	x	--
Quercus fabri	OAK-MOR-545	--	x
Quercus faginea	OAK-MOR-215	--	--
Quercus faginea	OAK-MOR-216	--	--
Quercus faginea	OAK-MOR-456	--	--
Quercus faginea	OAK-MOR-658	--	--
Quercus faginea broteroi	OAK-MOR-278	--	--
Quercus fusiformis	OAK-MOR-200	--	--
Quercus fusiformis	OAK-MOR-218	--	--
Quercus fusiformis	OAK-MOR-307	--	--
Quercus fusiformis	OAK-MOR-654	--	--
Quercus fusiformis (x Q. virg. ?)	OAK-MOR-312	--	--
Quercus gambelii	OAK-MOR-132	--	x
Quercus gambelii	OAK-MOR-198	--	--
Quercus gambelii	OAK-MOR-630	--	x
Quercus gambelii	OAK-MOR-635	--	x
Quercus lyrata	OAK-MOR-385	x	x
Quercus lyrata	OAK-MOR-611	x	x
Quercus (macrocarpa x gambelli)	OAK-MOR-300	--	x

Quercus macrocarpa	OAK-MOR-616	--	--
Quercus macrocarpa	OAK-MOR-283	--	x
Quercus macrocarpa	OAK-MOR-357	x	x
Quercus macrocarpa	OAK-MOR-615	x	x
Quercus macrocarpa	OAK-MOR-671	x	x
Quercus macrocarpa	OAK-MOR-279	--	x
Quercus macrocarpa	OAK-MOR-290	--	x
Quercus macrocarpa	OAK-MOR-249	x	x
Quercus macrocarpa	OAK-MOR-487	x	x
Quercus macrocarpa	OAK-MOR-614	x	x
Quercus macrocarpa	OAK-MOR-672	x	x
Quercus macrocarpa	OAK-MOR-673	x	x
Quercus margarettae	OAK-MOR-644	--	--
Quercus margarettae	OAK-MOR-223	--	--
Quercus margarettae	OAK-MOR-221	--	--
Quercus michauxii	OAK-MOR-353	x	x
Quercus michauxii	PM155	x	x
Quercus michauxii	OAK-MOR-694	x	x
Quercus michauxii	OAK-MOR-700	x	x
Quercus michauxii	PM143	x	x
Quercus minima	OAK-MOR-177	--	--
Quercus montana	OAK-MOR-352	x	x
Quercus montana	PM-76	--	x
Quercus montana	OAK-MOR-576	--	x
Quercus montana	OAK-MOR-575	x	x
Quercus montana	OAK-MOR-693	x	x
Quercus muehlenbergii	OAK-MOR-007	--	x
Quercus muehlenbergii	OAK-MOR-383	x	x
Quercus muehlenbergii	OAK-MOR-639	x	x
Quercus muehlenbergii	OAK-MOR-632	x	x
Quercus muehlenbergii	OAK-MOR-260	--	x
Quercus muehlenbergii var. brayi	OAK-MOR-164	--	--
Quercus oglethorpensis	OAK-MOR-548	x	--
Quercus oglethorpensis	OAK-MOR-549	x	--
Quercus petraea	OAK-MOR-072	--	x
Quercus petraea	OAK-MOR-229	--	x
Quercus petraea	OAK-MOR-263	--	x
Quercus petraea subsp. pinnatiloba	OAK-MOR-227	--	--
Quercus prinoides	OAK-MOR-143	--	x
Quercus prinoides	OAK-MOR-157	--	x

Quercus prinoides	OAK-MOR-532	x	x
Quercus prinoides	PM-93b	x	x
Quercus prinoides	OAK-MOR-005	--	x
Quercus robur	OAK-MOR-692	--	--
Quercus robur	OAK-MOR-082	--	x
Quercus robur	OAK-MOR-190	--	--
Quercus robur	OAK-MOR-122	--	x
Quercus sinuata	OAK-MOR-418	x	--
Quercus sinuata	OAK-MOR-448	x	--
Quercus sinuata var. breviloba	OAK-MOR-185	--	--
Quercus sinuata var. breviloba	OAK-MOR-236	--	--
Quercus sinuata var. sinuata	OAK-MOR-187	--	--
Quercus stellata	OAK-MOR-074	--	x
Quercus stellata	OAK-MOR-340	x	x
Quercus stellata	PM11	x	x
Quercus stellata	PM145	x	x
Quercus stellata	OAK-MOR-194	--	x
Quercus stellata	OAK-MOR-251	--	x

Supplementary file 2. Custom bash script for SNP detection after PyRAD/RADami analysis.

```
#!/bin/bash
# TL-13/01/15
# Create files per sites and per locus
while read line; do
      echo "$line" > tmp
      a=$(awk '{print $1}' tmp)
      b=$(awk '{print $2}' tmp)
      IFS=', ' read -a array <<< "$b"
      for element in "${array[@]}"; do cat
            while read line; do
                  echo "$line" > tmp2
                  if [[ "$line" == ">"* ]]; then
                        d=$(echo $line)
                  elif [[ "$line" != ">"* ]];
                  then
                        c=$(cut -c "$element" tmp2)
                        echo "$d   $c" >> $a.$element.output
                  else
                        continue
                  fi
            done < $a
      done
done < liste
```

Supplementary file 3. Custom bash script for SNP detection after STACKS analysis.

```
#!/bin/bash
while read line;
      do echo "$line" > tmp
      a=$(awk '{print $3}' tmp)
      grep "$a  " sampleid.txt > tmp2
      awk '{print $2}' tmp2 > tmp3
      while read line;
            do c=$(awk '{print $1}' tmp)
            d=$(awk '{print $2}' tmp)
            e=$(awk '{print $4}' tmp)
            f=$(awk '{print $5}' tmp)
            echo "$c     $line      $b      $e      $f"
      done < tmp3
done < batch_11_p10r75sumstats_select.txt
```

Supplementary file 4. Barcode selection using phylogenetic approach via phylogeny.fr

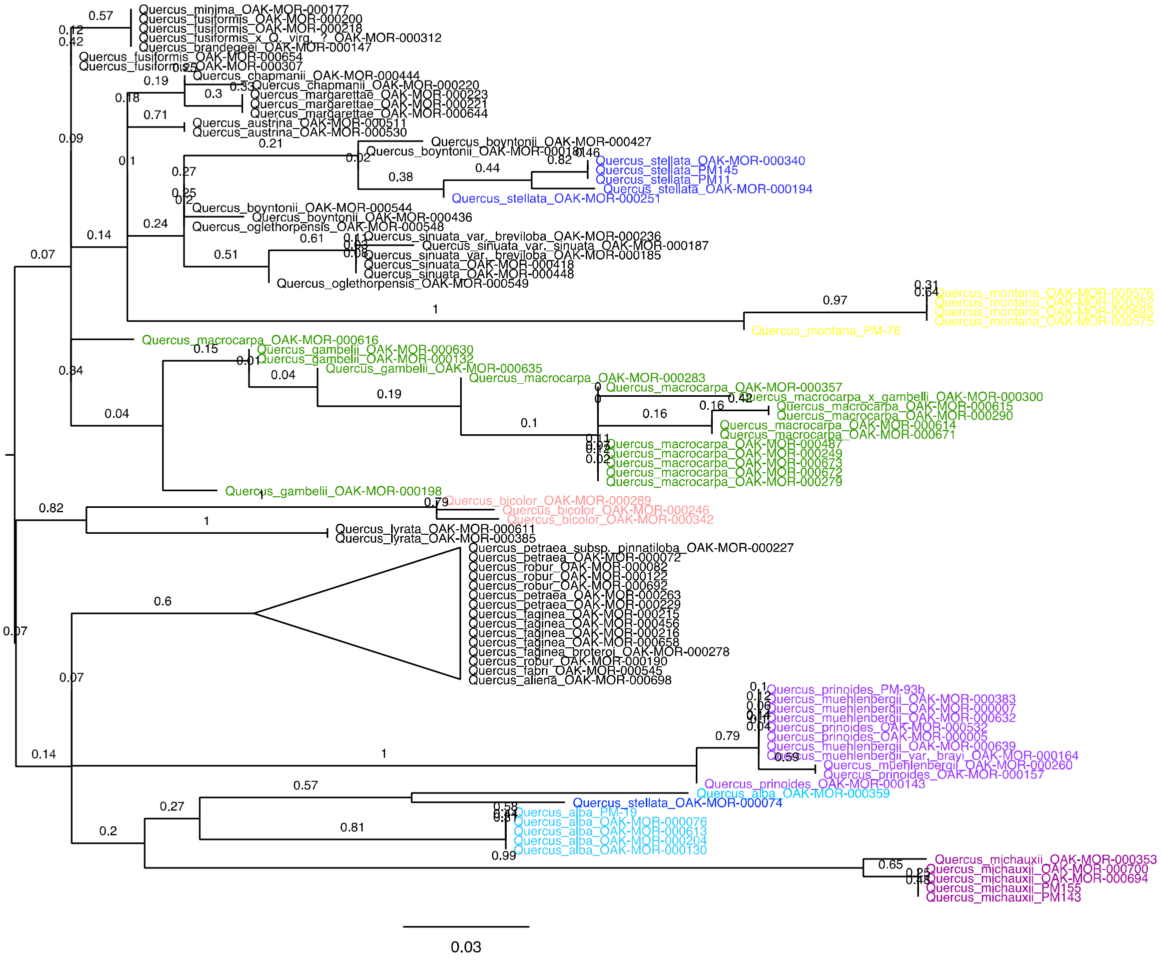

Supplementary file 5. List of 80 SNP-multiplex

Species	SNP_ID	Primer sequence
Q. alba	locus_10977_45	CATTGCCAAAAGGAGGTGCAGCAAAAATGACATCTCGAAGTGGA[G/A]GCTCAGCAAAACAGCTTTTTCCTGTATCTTCGAATTGC
Q. alba	locus_5882_32	TGGCATCACTTGAACCCAAAAATGGATTATG[A/G]TAATAGAAATTTTCTTCCAATTTTTATCCTTTTAAAAAGCATCATTTTTT
Q. alba	locus_821_26	ATGTTACTTGTGCCAACCCCACATC[T/C]AACAATTCCATCAACTCAAATTCAGTTAAAGATAATGCATCCTGAAAGCAAACAAC
Q. alba	CL_54979_37	TTAAGCCAGTCCCATCATTCAAGGCCGATGT[G/T]TATGCACTTGGGGTGATTCTAATGGAATTGTTAACCAGGAGAAGTGCGGGTGAC
Q. alba	CL_35240_32	GAATATGCGGATGTTGTGTTTCCAAG[C/T]TACTTCTCACCATTCCTGCATAATTTCTACTGCTACTGGAATCCTCTTCATTCTGATAT
Q. austrina	locus_11631_48	CAAGCTTTTAACCCAAGGATGCCCCAGTCTGTGGGTCAA[T/C]CTTCAAGCCCTATTCCACCATCTTCGCACCCCTCGCGGACCCA
Q. austrina	locus_20180_49	CTAGAGAATATCTTTTGATTTTTAGTTTAGAAGGGAAGCTAARGATTT[G/A]AAGGATGGCGGCTACCCTATGAATACTTTCAAGAAG
Q. austrina	locus_295_56	CAAATTAACAAAAGGGTATAAAAGAC--AGTTTTCAGTGTCTCTCTCTATTGACA[T/C]TCAATAGCACATTCTTGGCAGAGCTTAGA
Q. austrina	locus_3169_44	TAGAAGGCATAGCTTGCACTATTAAATCATCGATTTGATGTAT[A/G]AAGGGGTTAAACAAAAATGAAAGATTTTATGCATACCTTGT
Q. austrina	locus_5422_58	ATGGGATCCTTTTGGGAGGCATTCTGGTCATGTATGAGTTCCATAATTTTCTGAGAC[C/A]TATTCTTCTCCTTTTGTACTCTTTTT
Q. bicolor	locus_1378_30	ACTTTGATATGATGATCCAGATAGAATGC[A/G]GTAGAACAAGTTAATATTTTCAGAACCGCATCTGGAATCATCTCACCATTTACAA

Q. bicolor	locus_24383_42	AGAAAGCAGAAAATGAATAGTTAAAGCTGAAGTCAGGGTGA[T/A]GCCCTTTTTTGTTAAATTATT GGATAATTGGAAGATTGGTTTC
Q. bicolor	locus_26761_43	CGTAATTTTGAAGTT-ACAACACATCATGGCCTGCATTGGAC[A/G]CAATCATCTG-- GTTGCTCAAATTTGGTCCAGTATA-AT
Q. bicolor	locus_461_36	GTTTATCATTCCCCCATCTGGCCACAATTAATAGA[T/C]GGAGTTATTCAAATGTTTAACCTATTCTT CATACCTAGACAAACAAGAC
Q. bicolor	locus_4850_29	CAATGTTGGCAAAGTATTCTGATCCATT[G/A]GTATATACAGGACCTATACGGGTGAGAACAGGCC ATGAGATCTTGCGTATTTCTTC
Q. chapmanii	locus_2684_26	AATCGCAGATGGGCTTGGCGGCTTT[C/G]AATGACGGTGTCCAATTTGGCCCAAGTTATTTGGGTC ATCTTATTCA-CCGGATTTAGG
Q. lyrata	locus_3962_56	AGCCTTGGAGGATCTTGATTCCTTACTTCTAAATGCTTCAAGAAATGATCCAGGA[G/A]CCTCAGTT GAATCAATGAAAGGAAAGATC
Q. lyrata	locus_8059_35	GAAAGGAGTTCCATTAGGCATGTCTTTCCCCTGT[T/C]GCCTCTTCTTCCCTATCCCTAGCACCTGCT GAAGACATGCAGCAACCATA
Q. lyrata	locus_9121_49	AAGCCAATTTTTATAGCATAAAGATAACTGAGGACAGAATCAAAACCA[C/T]TT- CAAGTAAGATGACATATTATAAGAGATATAA
Q. lyrata	locus_8617_30	TTATCAACCCAAGAACAACAATCCTTGAA[G/T]AGG-AAAAAATAG- TTTTACTTTTATTTAATCGCTAATTCAATAATGAAACCTGCA
Q. macrocarpa	CL_49943_41	GCTTTTGTTGGTTCAACATACATTTGTGTACCTTT[A/T]TTGTTTTGAATTAAAGTAGTNTTTGACAT GGACAGGATTACAGCCTTTAG
Q. macrocarpa	locus_11302_50	AATTACCACAAATTTGAAGATATAAGGTCCCTTCTCTTTAACTATTTTC[C/T]GCCCATGGGTCTCA TACACCCTCTTCGGATCTTTA
Q. macrocarpa	locus_3999_44	TAGTTTTTTCTGCATAATGTTTTTGTTCTATAACCTTTATAAA[C/T]CCGTGTGTTGTGATTAATATA TGAGATATATTAATTGTTCC
Q. macrocarpa	newl_25158_45	CACCAGTCTTTCCTCTTGTTGTTGGGGTTTCAGTGGTTGCCTTG[G/T]TTATCAGTATGCCAAGGTAA TTGTAATATTATTTTCCCTA

Q. michauxii	locus_10809_46	GTCGGGTCCGGGCATAAAGAAACCCGACCCATTGCCATTCCTAAG[T/C]CTAGGAATCAACTACTCCAACAAACAATTGGGGGTAA
Q. michauxii	locus_13856_44	AACTTTTCAAGATGGTTTGTGAACATAATGAAACCGACCTAGA[T/C]ATAAAAATACCTACAGTAATGCTCCCACAAGATGCTGGT
Q. michauxii	locus_17927_52	CATAAAGTCTTTGCGAAGAATATGCCGCACACCATGCAATGCAGATGCTAC[A/G]GCATATGCATACCTTAATTTGACAAAGAAATG
Q. michauxii	locus_28072_46	TAAAATTTTCTGCATATTTATGTTTTAATCGTCGTTTACGATGTG[G/A]ATATGTTTAGAGATTCGTGGCATTTGTTTTAAATGGGAC
Q. michauxii	locus_31722_39	GAACACCATCTTCTTCAGGTAC-AAAATTTGAGTCCAA[C/A]TTACATTAGTTCCATACTGGAATAAAAATTGGTCCATTCTCATAATA
Q. michauxii	locus_7123_50	CAGAAAGTACATAAGACTTGTTAAATGTAATAAACTACAAAGTCAAAAG[C/G]TTGCCCCAAGAATGCATGTTGCAAGAAAGAATACC
Q. michauxii	locus_7834_43	CACATGGACAATTTTAAGGCCCAATGCATCAAGCTGAGCACG[G/A]AACTGAGAAAGATCAGTTGGCTTTCCTTGCTTTTTGACC
Q. michauxii	locus_8226_51	TTGTACATTGCCTTGTTTCAGGTTGTTGATAAACCGTTTGAAAGTC--GT[C/A]TTTATTGACTTCTCGACACCTATCAT-AAAATCAT
Q. montana	locus_2085_53	ATCTAAGTCAAGAAACTCAAAGCTATGATTATGACATCCATCTTCCCACTGT[C/T]TACTTAACATGCAGCTCCAGGCCTAGTAAA
Q. montana	locus_26885_29	CCTGGCTATCATCTAAAACGGGTTGGAC[C/T]TCATTCCTTTTCTTGTCAGAAGCTTGGTCAAATTTCTGTACACATATATTCTG
Q. montana	locus_27743_25	TCTAAGGAATCGCAAGTACTTCCT[T/A]GATTTTCCCTACACATGAAATACCAAAATCATAAGTAAATTCCTGTTGAAGACTAATC
Q. montana	locus_28457_43	AACATATATAATAGTATAATACAATCATTCTGCGTTATTTCT[T/A]TTTATTTTGCCTGGCTATGCCTAGAAAATAATCCAGGAGC
Q. montana	locus_30512_25	TGCCCTTTCCTTCATTCTCTCAGC[T/C]TTTTCTACTTCTTCTCTAGCTCTCTCCCACATGTGCCTTGCACGTGCGAACTCAGAC

Q. montana	locus_30948_43	TGTTGGGACATCCACGGGAGAAGAATAGGATGAAGAATATTA[G/A]TGGGAAGAGTTCAGGTAAT TCACCTCAAAAGAGTCAGCAAGG
Q. montana	locus_5482_34	CATCAGCTTCTATTATCTATACACCATTCATTG[C/T]ATTGTCAGGATGGCCTGTGTTGCCATCTTCC AGATTCTTGCCGTTGAC
Q. montana	newl_21880_27	ACTGGACTGGCTGCACCATAAATTTC[G/A]TCATAATAGCATCGAGCCTTTCAAGGATTTTACAATT GGTATTCAAACTGTCCTCA
Q. muehlenbergii/prinoides	CL_18142_42	AATTCTCTTTTCCTCTGGTTGCTTTTTGCCCTTTTG[G/A]TTTCCTTTTCCTTTCCCCCTTAGATTCGA AGAAAAATCACTGTTTAGTT
Q. muehlenbergii/prinoides	CL_42027_49	TACAAGATCACATATTCATTTCAAATGAATNATAAAGAACATN[T/C]TAAACTATTCAACAGTAGC AGCTCTCATGATTCAAAGAAACC
Q. muehlenbergii/prinoides	CL_48165_56	TTGCTTATATCATCAGGAATATGGCCAACAAGGTGGAGATTGTGCAAATC[C/T]AGAATGAGAAGC AGCTCTATGCTTCCCAACTCCTT
Q. muehlenbergii/prinoides	locus_10104_41	AAGGAGATGAACATTCATACTGCATCTTCAGTTGTTCTTG[T/C]AGTTGACTTTGCTCTAGTCTATA TGACATGTCATGATTTTCCTG
Q. muehlenbergii/prinoides	locus_12538_49	ATTCAGGTTCTTTGCCTTCTCTATTG--- CATCATCTATCCCAGGGAA[G/C]GTTTTAGATCCATAGTCATCGTGCTTTGAGGGCCCATA
Q. muehlenbergii_prinoides	locus_29214_32	TCAGGCCTTATGATGGTAATTATTGTATGAA[C/T]TGTCCACCGTTGTGTTTACCATTTTGTTATCTT TTATAAATGAAACATTAGC
Q. muehlenbergii/prinoides	newl_17339_35	CGAGTNATAGCTTCGCTAATAGAACATATGGCNT[C/T]ATCTTGCCAGCAAACCTTTTNAGCAAGA AATGTCCTAAGGGATTTAGGAT
Q.	newl_20204_33	CTGTTCTGTAAATGTTATGTGGTTGACTTGAA[A/G]GATGAAAATTCTTGTAAATGCATGTATNATA

muehlenbergii/prinoides		CAGGATTAATAATATGCCTT
Q. oglethorpensis	locus_16087_43	CCTTGGTACGTTTTTGAAAATTAAATTATCCAGTGGACCTAA[A/G]CGAGCTTAGTCTGTCTGCCAATCATGGCATCA-TAGACACTA
Q. oglethorpensis	locus_32208_54	TCCCTTCAGTGCAATTTTGATTCAATAACTGCAACAAATTAGTAATCTTA-TA[A/G]TAATTGGCTAGAATGCATGTCAATCAGTGGGT
Q. oglethorpensis	locus_4100_37	AAATTTATTAGCTTCTGCAATCACGAAACGACTGAT[G/T]CCCAAGTAAAAGCTCAGGTTGGAAAGAGATGAGAAGTAGGAATTGG
Q. oglethorpensis	locus_4492_52	AACAAAAGTT---GAAGACGGGTAGAGTGCAGCACAATAAATTTCTTGCTT[A/G]TCTTTATCAAGTAATGACAGTGGAATTACAAGT
Q. sinuata	locus_10802_36	AATCTTTCTTACTGACATTGACATATCTGTTATCA[G/A]TTTCATCAGACTTGNCTCCAAACTTCTCCTGTAACTGGTTTTTACCAT
Q. sinuata	locus_20667_37	TCGGAACAATAGGCAAAGTGGGCAAGAATTTGGCAA[C/T]AAAAGAGAGAACCTCAGGTATTGGCCATTTGGGTCTCACAATATCAGA
Q. sinuata	locus_23517_52	AACCAGCGGGTCGAATTTCCAATAAAGTCCAACTTGTAAGGCTTATCTGCC[C/G]TGGTCAGACCTAGACTGACCCATATTTC-TGT
Q. sinuata	locus_27412_25	ACGAAGTTTGCAAGGCGTGGCTAC[T/A]AGGGAAGCTCCCAAGGGCCATGATGATTTGTTTACACCAAACCAAGAACATGAAGAGG
Q. sinuata	locus_5229_56	TGAGCCACTTAAAAATGGGGACCAATGAGGGAGTGAGGAAAAATAGAGCACTGCT[G/A]GTTTGGATTTCGATTCTAAGGGCTAAGGC
Q. sinuata	locus_792_52	AACCAGGTAACATTAACAAATTAAGGTTCTGTTGCTGCAATGTAAATATGT[A/C]ATTTTTGAATGTACAGGTAGTGAAACAGTGCC
Q. sinuata	locus_8717_53	TAATAGCTCAATATTGTGGACAAGCAGCGTACCTCAGGAAATTCCCAGAGAA[T/C]GTGTCTAATACGTTCTACGACTCCATACCAGG
Q. sinuata	locus_9837_55	TGTTGCATCCCAAATGTCCAACTAGCTANATGCCCATGCTTATCTATGAGAAGA[C/A]CTGAGCTAT

		C-AAACTCAGGCCTCTTCTCA
Q. stellata	CL_11069_58	CAAGTCACATTGCTAAAGTTGAGGATGATTTCCATGAAATGCAAGANCTGAN[A/G]GTTCGTGCAG AAGCTGCTGACGTTGCAAAATCT
Q. stellata	CL_12923_48	GAGAGCAGAATCTGGTTCAATGGGTAAGAGACTGCCACCTTA[A/G]TTTCATATTTCATGTACTGA TGTTGATAGGGGTTCCCGGCCTT
Q. stellata	CL_24297_67	ATGAGGTACCCGATTATACCACGACNACAATTCAAGGGACCAAGGTGAAGATATTTGATTA[T/C]G ATGAAGAGGTGGAGATAGTGTTT
Q. stellata	CL_2457_66	CAATATCCTCTGCACGCTTCCTCGCCNCTTCAGCTTTTCTGGCTGACTCATCAGCATCCT[A/G]CATT AAAGTACATATGTATTAAGAA
Q. stellata	CL_2457_32	CAATATCCTCTGCACGCTTCCTCGCC[A/G]CTTCGGCTTTTCTGGCTGACTCATCAGCATCCTNCATT AAAGTACATATGTATTAAGAA
Q. stellata	CL_45_63	CAACTGATACGTGGTCAGATTGGCATAATACNTCTGTGCCACCGTCAAGTACGTATC[C/G]CCTGA ATGAACCGTGTACTCAAACATGT
Q. stellata	CL_49075_43	GCTGAGTTGCTTCTAAAACTTCCTCCAGCTCACCTAG[G/A]AACATAACCTCCTTAGAACTATTCGT AATTGGCCAATATTTTAGTAAA
Q. stellata	CL_55087_32	CGACCAAGCTACAATGCTTCAGGGTA[T/G]TCTCTCTCCTGCTATACTAATGTTGCACTTCCTGTCC TTGCAATTCGTGTTAAATGTTC
Q. stellata	CL_6426_61	TAGCTTCACATAGCAAGAGTCTGCTCGTTTGCTCAGCTGAACCAACGACCTGATC[A/G]AGGTTTC AATATAACAAGGTTAATTCAGAA
Q. stellata	locus_14289_31	ATTTGTCCTCGTGGGTAAAGCTTCGGAACA[C/T]GCTCACTATAGAAAAATCTTTACCTTATTTGGG ACCCCCATCTTCCATGTACG
Q. stellata	locus_17368_30	GATTGATGGTTTCATGTGACCCAAAGTAA[A/C]AAACTGCCCTTAGCCCCACCATATGATGTGGGT CATCAACTTGTGAACTTGTGT
Q. stellata	locus_8104_38	TTTTGGGGGTCAAAGAGTTCATAGTCCTACTCCAC-- [T/C]ATGCAGTGTAGACTGTAGGGTGGGGTGAAGGGGTTGGAAATATGCACAT

Q. stellata	newl_15918_41	GTTGATACTTATCTGAGTGCAAGAGGGNCCCAACCATGAN[C/T]TTGTTGTTTCTGGCATCAAATTTGTCTATGTAATCATTGATTTTT
**East Asian white oaks**	newl_15225_27	ATCAAGGAGGGCTGCTTTCTTTGTGT[A/G]GGACGGCCTGTTGGGGAGAGATCTTGAMCAATGAAAATCTTCAAAAAAAGAGGTATC
**East Asian white oaks**	newl_16979_31	CTACAGTAGAGGCTATTTTAGTGCCAAGCA[A/G]ATAGACCTTATTAAAGTCTCACTAATGGAAGCAACACCCCCTCGACCCTAGATC
**East Asian white oaks**	newl_23554_43	ATCAACGCCATCATTCCTTTCTGCKRGTAGRGTATTACCATG[C/T]TTTTTGTGCCACACAAGGATCCGCTTTCTTGCAATATCACA
**East Asian white oaks**	newl_27648_32	ACAACCCATTCATCCTGCAATAAAACGTACA[A/G]TTAAYATCATTCAATTTACYRTCTGTCTTCAATTAATTAGTTAAGAGGTAT
**European white oaks**	newl_28982_61	TGAGCCTGCTGTGATAGAGATNNNTNCCCCCCCAGAAGCTGTTATTGTGCCAGTTGCACT[G/A]CTTGGGGTTTTAGATCACCCAATATA
**Clade 1**	CL_31994_43	CATCAGCATTTTGGTATCATGGATCAACTCACTCCCA[T/C]GACTTAACCCAATTAGGTAGTGCTGCTGAAGTCCAAAGCCGTGAAACC
**Clade 1**	locus_25236_45	TTTCTGTTTATCTACTTGTCATCTCTTTTATTCTTATTATTTAT[G/T]ATTTAATCTTATAAACACTATCCTAAACAAGAGATGATTC
